# *Trpm4* Gene Invalidation Leads to Cardiac Hypertrophy and Electrophysiological Alterations

**DOI:** 10.1371/journal.pone.0115256

**Published:** 2014-12-22

**Authors:** Marie Demion, Jérôme Thireau, Mélanie Gueffier, Amanda Finan, Ziad Khoueiry, Cécile Cassan, Nicolas Serafini, Franck Aimond, Mathieu Granier, Jean-Luc Pasquié, Pierre Launay, Sylvain Richard

**Affiliations:** 1 INSERM U1046, Université Montpellier1, Université Montpellier2, Montpellier, France; 2 Equipe Avenir, INSERM U1149, Université Paris 7, Paris, France; 3 CHRU Montpellier, Service de Cardiologie, Montpellier, France; Georgia State University, United States of America

## Abstract

**Rationale:**

TRPM4 is a non-selective Ca^2+^-activated cation channel expressed in the heart, particularly in the atria or conduction tissue. Mutations in the *Trpm4* gene were recently associated with several human conduction disorders such as Brugada syndrome. TRPM4 channel has also been implicated at the ventricular level, in inotropism or in arrhythmia genesis due to stresses such as ß-adrenergic stimulation, ischemia-reperfusion, and hypoxia re-oxygenation. However, the physiological role of the TRPM4 channel in the healthy heart remains unclear.

**Objectives:**

We aimed to investigate the role of the TRPM4 channel on whole cardiac function with a *Trpm4* gene knock-out mouse (*Trpm4* ^-/-^) model.

**Methods and Results:**

Morpho-functional analysis revealed left ventricular (LV) eccentric hypertrophy in *Trpm4* ^-/-^ mice, with an increase in both wall thickness and chamber size in the adult mouse (aged 32 weeks) when compared to *Trpm4^+/+^* littermate controls. Immunofluorescence on frozen heart cryosections and qPCR analysis showed no fibrosis or cellular hypertrophy. Instead, cardiomyocytes in *Trpm4^-/-^* mice were smaller than *Trpm4^+/+^*with a higher density. Immunofluorescent labeling for phospho-histone H3, a mitosis marker, showed that the number of mitotic myocytes was increased 3-fold in the *Trpm4^-/-^*neonatal stage, suggesting hyperplasia. Adult *Trpm4* ^-/-^ mice presented multilevel conduction blocks, as attested by PR and QRS lengthening in surface ECGs and confirmed by intracardiac exploration. *Trpm4^-/-^*mice also exhibited Luciani-Wenckebach atrioventricular blocks, which were reduced following atropine infusion, suggesting paroxysmal parasympathetic overdrive. In addition, *Trpm4* ^-/-^ mice exhibited shorter action potentials in atrial cells. This shortening was unrelated to modifications of the voltage-gated Ca^2+^ or K^+^ currents involved in the repolarizing phase.

**Conclusions:**

TRPM4 has pleiotropic roles in the heart, including the regulation of conduction and cellular electrical activity which impact heart development.

## Introduction

Transient Receptor Melastatin 4 channel (TRPM4) is a Ca^2+^-activated non selective cation channel permeable to monovalent cations (Na^+^ and K^+^) [1], [2]. Studies in mice with a deletion of the *Trpm4* gene (*Trpm4* ^-/-^) have shown that TRPM4 corresponds to the Ca^2+^-activated non-selective cationic current (NSC_Ca_) in different tissues including mast cells, dendritic cells and cerebral arteries [3]–[5]. This current is also present in murine sino-atrial node cells and in human atrial cardiomyocytes corresponding to robust expression of TRPM4 in the conduction system and atrial cells [6]–[8]. In contrast, neither the TRPM4 channel nor the NSC_Ca_ current are hardly detectable in rat or murine freshly isolated ventricular cardiomyocytes [9]–[11].

The physiological role of the TRPM4 channel in cardiac function has been investigated in the *Trpm4^-/-^* mouse or in mice treated with 9-Phenanthrol, a TRPM4 specific inhibitor.

Deletion of the *Trpm4* gene causes markedly more acetylcholine-induced exocytotic release events leading to hypertension [12]. In*Trpm4^-/-^* ventricular cardiomyocytes, the Ca^2+^ transient may be increased during excitation-contraction coupling under β-adrenergic stimulation [13].

In the atria, TRPM4 channel blockade by 9-Phenanthrol shortens the action potential (AP) duration suggesting that TRPM4 delays AP repolarization [14] whereas it has no effect in the ventricle. Moreover, application of 9-Phenanthrol can reduce the rate of spontaneous atrial beats, suggesting a role of the TRPM4 channel in sino-atrial node AP triggering [15].

Two different studies have also shown a cardioprotective and an anti-arrhythmic effect of 9-Phenanthrol after ischemia-reperfusion and hypoxia re-oxygenation, respectively, suggesting that TRPM4 is likely involved in the response to these stresses [16], [17].

Recent literature has reported that human *Trpm4* gene mutations generate conductions disorders such as right bundle branch blocks or Brugada syndrome. The first mutation described is a c.19G→A missense mutation, which results in the modification of the N-terminal protein sequence and promotes a dominant gain of channel function. The molecular mechanism at work involves an elevated density of TRPM4 at the membrane level [8] due to impaired deSUMOylation, an important step for channel protein degradation. A mutated channel in humans expressed in heterologous systems is however difficult to transpose on conduction tissue function. Moreover, in the Brugada syndrome, both gain of function as well as loss of function of TRPM4 channel has been described [18]. In both cases, it is unknown how the modifications can transform the physiological role of this channel which to participate to this syndrome.

Based on the current literature, TRPM4 may i) act as a calcium regulator, ii) influence cardiac conduction when overexpressed and iii) play on AP duration in the atria as well as in the ventricle in physiological conditions. However, the lack of TRPM4 channel (Trpm4 gene deletion as well as pharmacological tools) on AP duration has induced divergent results, particularly in the ventricle [13], [14]. All of the discrepancies reported could be partially explained by the heterogeneity of the study designs (human *vs.* mouse, isolated cells *vs.* tissue).

In this study, using a *Trpm4* gene knock-out mouse model (*Trpm4^-/-^*) [3], we investigated the consequences of a loss of TRPM4 function on adult cardiac morphology and function. We analyzed cardiac structure and contractile performance by echocardiography in adult *Trpm4^-/-^* mice. We also examined in vivo and in vitro electrophysiological properties compared to wild-type animals. We observed that increased hyperplasia in *Trpm4^-/-^* mice during the neonatal stage influences the adult left ventricular mass resulting in eccentric cardiac hypertrophy. We also demonstrated that *Trpm4^-/-^* mice exhibit potent conduction blocks, due to increased parasympathetic tone, as well as ectopic atrial activity, which have not been previously reported. Finally, we validated the direct functional involvement of the TRPM4 channel in the atrial but not ventricular AP waveform in resting conditions.

## Methods

### Animals

Knock-out mice (*Trpm4* ^-/-^) and littermate controls (*Trpm4* ^+/+^) were obtained as described [3]. Experiments were performed on 12 and 32 week-old male mice (see Table 1). All procedures conformed to the Directive 2010/63/EU of the European Parliament and the Council of 22 September 2010 on the protection of animals used for scientific purposes (agreement number: A34-172-38), and was approved by the comité Ethique pour l′Expérimentation Animale - Région Languedoc-Roussillon (protocol number: CEEA-LR-12110). Mice were housed in a pathogen free, controlled environment (21±1°C; humidity 60%; lights on 08:00 AM - 8:00 PM; food and water available *ad libitum*; enriched environment) with5 mice per cage. In ECG experiments mice with telemetric device were isolated in individual cages for recordings. All efforts were made to minimize animal suffering and where appropriate, mice were anesthetized with isofluorane (echocardiography) or Etomidate (intracardiac electrophysiological exploration). The NC3Rs ARRIVE Guidelines checklist is presented in the S1 Checklist.

**Table 1:** Evolution of the weight of the mice with age.

Mice weight	*Trpm4^+/+^*	*Trpm4^-/-^*
12 weeks old	26.8±0.87g (n = 7)	24.7±0.49g (n = 6) (ns)
32 weeks old	28.19±0.69g (n = 8)	35.1±0.84g (n = 7) (**)

** *P*<0.01.

### PCR

Genomic PCR was performed on tail DNA with primers (Sigma-Aldrich) specific for the wild-type and null alleles (as described [3]). Total RNA was isolated from a minimum of 5 samples per group using a Nucleospin total RNA isolation kit (Macherey-Nagel, Hoerdt, France) according to the manufacturer' instructions. Total RNA, oligo-dT and random hexamer primers were used to generate cDNA using a Verso enzyme kit (ThermoFischer, Illkirch, France). RT-PCR for the evaluation of the expression of *Trpm4*, *Gapdh*, *Rps14, Connexin 30.2, Connexin 40*, *Connexin 43*, *Connexin 45*, *Collagen 1*and *Collagen 3* was performed using gene- specific primers and performed in duplicate. Reactions were achieved using SYBR green Mix (Roche Applied System, Meylan, France) and commercially prepared primers (Sigma-Aldrich, Saint-Quentin Fallavier, France) (see primer sequences in Table 2).

**Table 2:** Primers sequences for quantitative PCR.

Gene Name	Sense Primers (5'–3')	Anti-sense Primers (5'–3')
***Trpm4***	GAGAAGCCCACAGATGCCTATG	AGCACCGACACCACCAAGTTTG
***Rps14***	TGGTGTCTGCCACATCTTTGC	TTCATTCTGCCAGTCACTCGG
***Gapdh***	CTTCACCACCATGGAGAAGGC	GGCATGGACTGTGGTCATGAG
***Cx 30.2***	AGCAGGAGGAGTTCGTGTGT	TGGAAGAGCCAGAAGCGGTA
***Cx 40***	CCAAACCAGGAGCAGATTCC	GCGTACTCTGGCTTCTGGCTAT
***Cx 43***	ACAAGTCCTTCCCCATCTCTCA	GTGTGGGCACAGACACGAAT
***Cx 45***	ACAGTAAAAGGAGGGAACTTGATGA	GGGTGTGTTCCAAGTGAAAGG
***Coll 1***	TGGTACATCAGCCCGAAC	GTCAGCTGGATAGCGACA
***Coll 3***	GACAGATTCTGGTGCAGAGA	CATCAACGACATCTTCAGGAAT

For *Trpm4* gene expression comparison, we used two housekeeping genes in accordance with the developmental stage of samples (*Gapdh* for adult samples and *Rps14* for neonate samples, except for SAN tissue, where both housekeeping genes were used). Each sample was then compared to SAN (SAN (n = 4) *vs.* P1 (n = 6), using *Rps14* housekeeping gene, or SAN *vs.* RA (n = 6), LA (n = 6), Septum (n = 6), LV (n = 6) and RV (n = 6), using *Gapdh* housekeeping gene). We analyzed LA and LV from 4 *Trpm4^+/+^* and 5 *Trpm4^-/-^* mice, respectively for the expression of all connexin genes. Collagen 1 and 3 expression was evaluated on LV from 4 *Trpm4^+/+^* and 3 *Trpm4^-/-^* mice, respectively.

### Echocardiography

Echocardiography was performed using a Vevo 2100 ultrasound system (VisualSonics, Toronto, Canada) equipped with a real-time micro-visualization scan head probe (MS-550D) operating at a frame rate ranging from 740 frames per sec (fps). Researchers were blinded during echocardiograms recordings and analysis. Recordings were performed during one day for each series, with *Trpm4^-/-^* and *Trpm4^+/+^*mice randomly selected.

The nosepiece-transducer used has a central frequency of 22 to 50 MHz, a focal length of 12.7 mm and 55 mm of nominal spatial resolution. The Vevo 2100 is equipped with ECG-gated kilohertz visualization software (EKVTM), which synthesizes high temporal resolution B-Mode images by combining several ECG-synchronized heart cycles. The EKV image reconstruction software produces B-mode sequences at up to 1000 frames per second.

Mice were anesthetized with isoflurane (IsofloH, ABBOTT S.A, Madrid, Spain) at a concentration of 3.5% for induction and between 1 to 1.5% for maintenance during the analysis with 100% Oxygen. Each animal was placed on a heated table in supine position with extremities tied to the table through four electrocardiographic leads. The chest was shaved using a chemical hair remover (Veet, Reckitt Benckise, Granollers, Spain). Ultrasound gel (Quick Eco-Gel, Lessa, Barcelona, Spain) was applied to the thorax surface to optimize the visibility of the cardiac chambers. The heart rate (HR) and respiratory rate of mice were recorded during the echocardiographic study. Two mice were excluded from the study due to very low HR.

Echocardiograms were acquired at baseline. Left ventricular (LV) characteristics were quantified according to the standards of the American Society of Echocardiology and the Vevo 2100 Protocol-Based Measurements and Calculations guide, as described in the following paragraphs.

LV diameters were measured on a two-dimensional (B-mode) parasternal long axis and short axis view.

The functional parameters of the heart were evaluated based on LV diameter measurements.

Left Ventricular End-Diastolic Volume (µL):
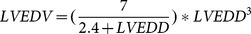



Left Ventricular End-Systolic Volume (µL):
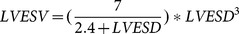



Stroke Volume corrected (µL/g):
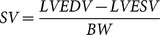



Fractional shortening (%):
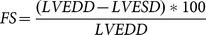



Left Ventricular Ejection Fraction (%):
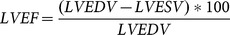



Cardiac output corrected (m(L/min)/g)  = 
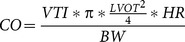



LV mass corrected (mg/g):
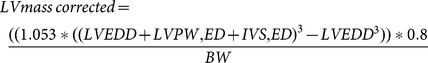



*BW: Body Weight*

*LVOT: Left Ventricular Outflow Tract length*

*VTI: LVOT Velocity Time Integral*

*HR: Heart Rate*

The diastolic relative wall thickness:
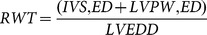



RWT was calculated as an index of cardiac remodeling (IVS, ED: End-Diastolic InterVentricular Septum thickness; LVPW, ED: End-Diastolic Left Ventricular Posterior Wall Thickness; LVEDD: Left Ventricular End-Diastolic Diameters).

### Immunolabeling

Mice were sacrificed by group in the morning by cervical dislocation. Hearts were quickly removed, rinsed and frozen in isopentane for 2 min. Adult heart (12 week old mice) cryosections were labeled with an anti-dystrophin antibody [19]and 4′,6-diamidino-2-phenylindole (DAPI, 1∶1000 from sigma-Aldrich), and examined using AxioVision Rel.4.8 software. Cell areas were calculated in 7 different sections per plane (transverse and longitudinal) from 3 and 4 independent heart samples for *Trpm4* ^+/+^ and *Trpm4* ^-/-^ mice, respectively. Approximately 150 and 500 cardiac myocytes were counted from longitudinal and transverse sections, respectively.

*Trpm4* ^+/+^ and *Trpm4* ^-/-^ heart sections from mice on postnatal day 1 (P1) were co-labeled with an anti-phospho-histone H3 antibody (1∶200, Cell Signaling, Saint-Quentin-en-Yvelines, France) and DAPI. Phospho-histone H3-positive myocyte nuclei and the total number of nuclei were counted in 4 different ventricular sections from 7 and 8 independent hearts samples from *Trpm4* ^+/+^ and *Trpm4* ^-/-^ mice, respectively. In atria, all positive nuclei from both auricles were counted in 3 mice per group. The percentage of mitotic cardiomyocytes was determined by the average number of phospho-histone H3-positive nuclei divided by the total number of nuclei. The *Trpm4* ^+/+^ hearts showed a percentage of mitotic nuclei close to previous report [20].

Samples were blinded during the labeling, imaging, and analysis of all sections.

### Electrocardiograms (ECGs)

For long-term ECG recordings in conscious and unrestrained 12-week-old mice, telemetry devices (model TA10ETA-F20, Data Sciences International St. Paul, MN, USA) were implanted under general anesthesia (2% inhaled isoflurane in O2, Aerrane, Baxter, France). ECGs were recorded using a telemetric receiver (model RPC-1) located below the animal cage and connected to a data acquisition system (IOX2, EMKA Technologies, Paris, France). Data were collected continuously over 24 hours at 2 KHz. Recordings were digitally pass-filtered between 0.1 and 1000 Hz and analyzed off-line with ECG-auto software, version 1.5.12.22 (EMKA Technologies). Twelve-hour nocturnal ECG signals were scanned by hand to detect, identify and count spontaneous rhythm disorders. RR, PR, QRS and QT intervals were measured. Heart rate variability (HRV) was analyzed in the time domain [21]. Ectopic beats were removed, assuming that the cardiac rhythm was the sinus rhythm. These beats were not replaced by any averaged or interpolated beat. The mean RR and the standard deviation of all normal RR intervals (SDNN) were calculated from 12-h ECGs. When atrioventricular blocks were detected, PR intervals were measured and HRV was analyzed based on the 6 QRS complexes preceding the block, to evaluate the involvement of the autonomic nervous system by calculating the square root of the mean square successive differences between successive normal intervals (RMSSD, in ms). For data collection, 3 *Trpm4^+/+^* and 3 *Trpm4^-/-^* mice per group were analyzed per time period due to equipment constraints. This procedure was then repeated on new groups until a total of 13 *Trpm4^+/+^*and 18 mice *Trpm4^-/-^*mice were analyzed.

To test the implication of parasympathetic activity in atrioventricular conduction delay, ECGs were monitored during atropine infusion (10 µg. kg^−1^. h^−1^ for 6 hours, Alzet 2001 micro-osmotic pumps implanted in the afternoon to avoid paradoxal effect of atropine). The mean RR and SDNN were then calculated, and the number of atrioventricular blocks counted over the 6 hours of parasympatholytic infusion. Three mice per group were used for this experiment.

Analysis of all ECGs was performed under blinded conditions.

### Intracardiac electrophysiological exploration

Mice (12 weeks) were anesthetized by intraperitoneal injection of Etomidate (Lipuro, 20 mg/kg, B. Braun Medical, Boulogne, France) and lidocaine hydrochloride was locally applied (Xylocaïn 0.5%, Laboratoires AstraZeneca, Rueil-Malmaison, France). All surgical procedures were carried out using a stereomicroscope (OPMI 1-DFC, Carl Zeiss, Jena, Germany). A surface ECG was monitored throughout the experiment through the subcutaneous placement of 29-gauge needles in approximate lead II of the Einthoven ECG to ensure good routing and placement of the catheter. Under sterile conditions, a 1.1 French octapolar catheter with an electrode spacing of 1 mm (EPR-800 Ultra-Miniature Catheter, Millar Instrument, Inc. Houston, Texas, USA) was introduced into the right atrium and ventricle via the right external jugular vein [22]. Body temperature was maintained with a warming pad at 36-37°C. Atrial, ventricular and His bundle electrical activities were recorded. Surface and intracardiac ECGs were recorded (ML865 PowerLab, ADInstruments Ltd, UK) and analyzed off-line with the ECG Analysis Module for Chart Software (ADInstruments Ltd, UK). Intracardiac ECGs were sampled at 2.0 kHz, amplified and filtered at 0.3 Hz to 1.0 KHz. Baseline cardiac cycle intervals were averaged from 10 consecutive PQRS complexes measured during four stable periods, including the resting sinus cycle length, RR interval, suprahisian (atrial-His or AH), and infrahisian (His-ventricular or HV) intervals (ms). Six mice per groups were used for this experiment.

Researchers were blinded during intracardiac recordings and analysis.

### Cellular electrophysiology

Atrial and LV myocytes were enzymatically isolated from *Trpm4^+/+^* and *Trpm4^-/-^* mice (12 weeks) sacrificed by cervical dislocation during the early morning for whole-cell patch-clamp recordings (Axon Instruments, Foster City, CA) at room temperature (22–24°C) for measurements of cell membrane capacitance, ionic currents and action potentials (AP) [23]. Cells were recorded within 6 hours of isolation.

APs were measured in the current clamp configuration in response to brief (1-2 ms) depolarizing current injections at 1 Hz. The resting membrane potential, the rate of rise (dV/dt), the amplitude and the duration at 20%, 50%, and 90% repolarization of the APs were measured (respectively, APD_20_, APD_50_ and APD_90_). In the voltage-clamp configuration, cell capacitances were measured following brief voltage steps (±10 mV) from a holding potential (HP) of −80 mV, to provide an estimation of cell size^3^.

Outward voltage-gated K^+^ currents were evoked by 4.5 s voltage depolarizing steps to potentials between −40 and +40 mV from a HP of −80 mV; voltage steps were presented in 10 mV increments at 12 s intervals. Inwardly rectifying K^+^ currents, I_K1_, were recorded in response to 350 ms voltage steps to test potentials between −60 mV and −120 mV (in 10 mV increments) from the same HP.

I_Ca,L_ currents were elicited by 200 ms depolarizing steps to potentials between −60 to +50 mV from a HP of −60 mV (to eliminate any contaminant Na^+^ current or T-type Ca^2+^ current). Voltage steps were presented in 10 mV increments at 2 s intervals.

For K^+^ current and AP recordings, intracellular pipette solution contained (mM): KCl 120; EGTA 8; HEPES 10; MgCl_2_ 6.8; CaCl_2_ 3; ATPNa_2_ 4; and GTPNa_2_ 0.4 (pH 7.2). The bath solution contained (mmol/L): NaCl 130; KCl 4; MgCl_2_ 1.8; CaCl_2_ 1.8; HEPES 10; glucose 11 (pH 7.4). Calcium current (I_Ca,L_) were recorded using an intrapipette solution containing (mmol/L): CsCl 100; TEA-Cl 20; EGTA 8; HEPES 10; CaCl_2_ 3; ATPNa_2_ 4; and GTPNa_2_ 0.4 (pH 7.3). The bath solution contained (mM): TEA-Cl 140; MgCl_2_ 2; CaCl_2_ 1.8; HEPES 10; glucose 10 (pH 7.4).

The researcher was blinded during the electrophysiological recordings and analysis.

At least 3 mice per genotype were used for these experiments and the number of cells analyzed is reported in Table 3.

**Table 3:** Number of recorded cells for each type of current.

	*Trpm4^+/+^* atrial cells	*Trpm4^-/-^* atrial cells	*Trpm4^+/+^* LV cells	*Trpm4^-/-^* LV cells
I_K_ recordings	10	9	18	19
AP recordings	7	11	14	30
I_K1_recodrings	6	10	18	18
I_Ca_ recordings	11	13	14	17
Cell capacitance	10	11	24	26

### Analysis

GraphPad Prism and Origin software were used for in the analysis of each experiment. All data are expressed as means ± standard error of the mean (SEM). A Mann-Whitney U test was performed for comparisons between*Trpm4^+/+^* and *Trpm4^-/-^* mice. The effect of atropine infusion was evaluated using the non-parametric Wilcoxon signed-rank test. Statistical comparison was performed between isolated cells used in electrophysiology studies. For immunolabeling studies, statistical analysis was compared between the mean obtained for each mouse. A *P*-value of 0.05 or less indicated a significant difference between groups.

For supplementary Material and Methods, refer to S1 Supporting Information.

## Results

### TRPM4 deletion induces increased LV mass

**Figure 1:**
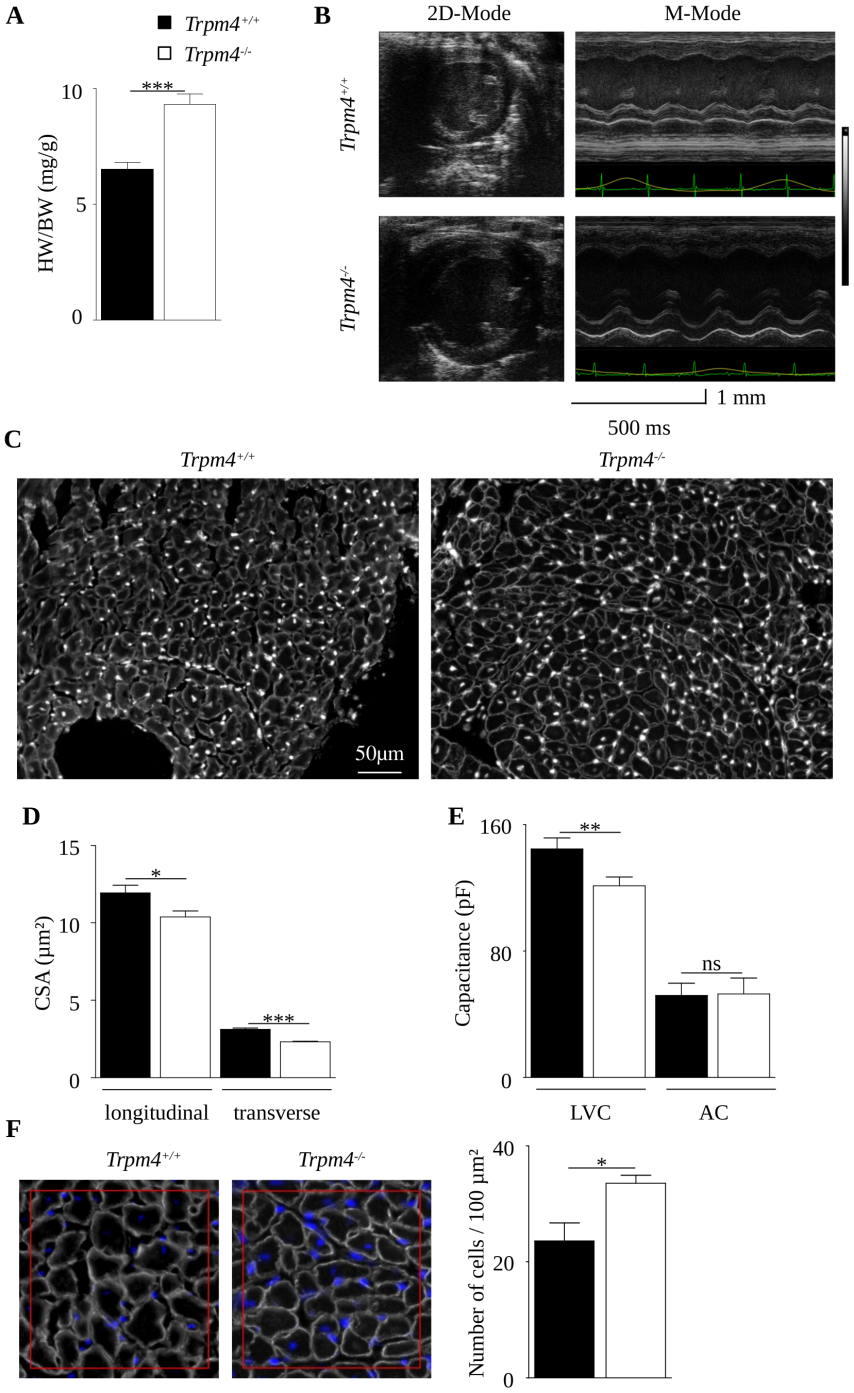
Increased left ventricular mass and cardiomyocytes densityin Trpm4^-/-^ mice. (**A**) Heart/body weight ratio (mg/g). (**B**) Parasternal short axis echocardiograms in B-mode (left panels), with images in diastole for Trpm4^+/+^ (upper) and Trpm4^-/-^ (lower)mice. Parasternal short axis view in M-mode (right panels) with images from Trpm4^+/+^ (upper) and Trpm4^-/-^ (lower) mice. Green and yellow traces in M-mode represent, respectively, ECG and respiratory activity during acquisition. Note the broadening of the QRS complex in the ECG from the Trpm4^-/-^ mouse. (**C**) Immunofluorescence labeling for dystrophin (grey) in longitudinal and transverse 12 weeks-old age adult LV sections counterstained with 4′,6-diamidino-2-phenylindole (DAPI) (white). (**D**) Histograms represent the mean cross section area (7 sections per mouse; 3 Trpm4^+/+^ and 4 Trpm4^-/-^ mice, respectively).(**E**) Cell capacitance of cardiomyocytes from the left ventricle (LVC, n = 24 for Trpm4^+/+^; n = 26 for Trpm4^-/-^) and from the atria (AC, n = 10 for Trpm4^+/+^; n = 11 for Trpm4^-/-^).(**F**) Magnification of images from (C) under a 40X objective showing cell density in 100 µm^2^ red square (DAPI is blue). Histogram represents the mean cell number per squares. Data are expressed as the mean ± S.E.M. *: *P*<0.05,**: *P*<0.01, ***: *P*<0.001.

*Trpm4* ^-/-^ and *Trpm4* ^+/+^ mice at 12 weeks of age had similar body weight (BW). However, the heart weight to BW ratio was higher in *Trpm4* ^-/-^animals (9.29±0.44 *vs.* 6.5±0.28 a.u. in *Trpm4^+/+^* mice, n = 6 and 5 respectively) (Fig. 1A) indicating cardiac hypertrophy. Echocardiography confirmed cardiac hypertrophy as *Trpm4* ^-/-^ mice exhibited an increase in left ventricular mass (LVM corrected and normalized to BW was 4.10±0.63mg/g, n = 6 *vs.*3.06±0.1, n = 7 in *Trpm4* ^+/+^ mice, *P*<0.01, Table 4 and S1 Supporting Information). On a functional level, however, *Trpm4* ^-/-^ mice showed no change in the LV fractional shortening (Table 5), consistent with preserved ventricular contractility. To determine if the LV hypertrophy in *Trpm4^-/-^* mice could be a first step to dilated cardiomyopathy (DCM), we followed the mice over time by echocardiography. At 32 weeks old, cardiac hypertrophy persisted in *Trpm4^-/-^* mice and was associated with diastolic dilation (S1 Table). Interestingly, *Trpm4* ^-/-^ mice still displayed preserved cardiac function as determined by fractional shortening, stroke volume and cardiac output normalized to BW (Fig. 1B and S2 Table). The relative wall thickness (the sum of anterior and posterior wall thicknesses divided by the ventricular diameter in diastole) was similar between *Trpm4^+/+^* and *Trpm4^-/-^*mice (S1A Fig.) indicating the development of eccentric hypertrophy.

**Table 4:** Left Ventricular functional parameters in 12 weeks-old Trpm4^+/+^ and Trpm4^-/-^ sedated mice.

	*Trpm4^+/+^*	*Trpm4^-/-^*	*P value*
Parameters	*n = 7*	*n = 6*	*12 weeks*
**LV mass corrected (mg/g)**	3.06±0.1	4.1±0.6	**
**LV EDV (µL)**	65.7±4.0	60.0±5.1	ns
**LV ESV (µL)**	32±3.3	28.3±4.8	ns
**Stroke volume corrected (µL/g)**	1.37±0.1	1.52±0.1	ns
**LVEF (%)**	62.5±5.5	64.7±4.4	ns
**LVFS (%)**	34.2±4.2	35.3±3.0	ns
**LVOT (mm)**	1.36±0.05	1.44±0.1	ns
**CO corrected ((mL/min)/g)**	0.53±0.03	0.55±0.05	ns

Values are mean ± SEM. LV mass corrected: Left Ventricular mass corrected; LV EDV: Left Ventricular End-Diastolic Volume; LV ESV: Left Ventricular End-Systolic Volume; LVEF: Left Ventricular Ejection Function; LVFS: Left Ventricular Fractional Shortening; LVOT: Left Ventricular Outflow Tract; CO: Cardiac Output; ** *P*<0.01.

**Table 5:** Left Ventricular basal characteristics in 12 weeks-old Trpm4^+/+^ and Trpm4^-/-^ mice.

	*Trpm4^+/+^*	*Trpm4^-/-^*	*P value*
Parameters (mm)	*n = 7*	*n = 6*	*12 weeks*
**IVS. ED**	0.77±0.02	0.95±0.03	**
**IVS. ES**	1.03±0.04	1.27±0.05	*
**LVEDD**	3.85±0.12	3.62±0.15	ns
**LVESD**	2.9±0.14	2.68±0.15	ns
**LVPW. ED**	0.7±0.05	0.94±0.04	**
**LVPW. ES**	0.91±0.06	1.22±0.06	**

Values are mean ± SEM. IVS, ED and IVS, ES: End-Diastolic and End-Systolic InterVentricular Septum thickness; LVEDD and LVESD: Left Ventricular End-Diastolic and End-Systolic Diameters; LVPW, ED and LVPW, ES: End-Diastolic and End-Systolic Left Ventricular Posterior Wall Thickness. ns, non significant,* *Trpm4^+/+^vs. Trpm4^-/-^*. * *P*<0.05, ** *P*<0.01, *** *P*<0.001.

The increased ventricular mass in *Trpm4* ^-/-^ mice may reflect a profibrotic phenotype as well as an increase of LV cardiomyocytes size. Histological tissue analysis using Goldner's trichrome staining, however, did not reveal signs of fibrosis (S1B Fig., n = 3 mice per group). Consistent with these results, the analysis of collagen mRNA expression showed that the expression of both collagen I and collagen III in the LV was similar in *Trpm4* ^-/-^ and *Trpm4* ^+/+^ mice (S1C Fig.), further supporting the idea that hypertrophy was not due to cardiac fibrosis.

We measured the cell surface area (CSA) of LV ventricular cardiomyocytes in cryosections of whole hearts by immunolabeling for the membrane protein marker, dystrophin. We found that CSA in both longitudinal and transverse planes were decreased in *Trpm4^-/-^* mice when compared to*Trpm4* ^+/+^mice (longitudinal: 1038±40 *vs.* 1194±49 µm^2^, *P<0.05* n = 156 and 170 cells for *Trpm4^-/-^* and *Trpm4^+/+^ mice,* respectively; transverse: 233±4 *vs.* 313±8 µm^2^, *P<0.001*n = 574 and 537 cells fo*r Trpm4^-/-^* and *Trpm4^+/+^* mice, respectively; Fig. 1C-D). To validate the decrease of cell size in *Trpm4^-/-^*mice, we used the patch-clamp technique to measure cell capacitance of freshly isolated LV cardiomyocytes. Cell capacitance directly reflects the cell surface area. These measurements confirmed the decrease in *Trpm4^-/-^* cardiomyocytes size compared to *Trpm4^+/+^* cell (121±6 pF, n = 36 *vs.* 145±7 pF, n = 24 in *Trpm4^+/+^*, *P*<0.01; Fig. 1E). In contrast, cell capacitance was unchanged in atrial cardiomyocytes (52.8±10 pF, n = 11 for *Trpm4^-/-^vs.*51.9±7.8 pF; n = 10, for *Trpm4^+/+^* mice; *P* = 0.8). Consistently with these results, cell densities measured over 100 µm^2^ cryosection areas were increased in *Trpm4^-/-^* mice (34±1 cells/100 µm^2^ *vs.*24±3 cells/100 µm^2^ in *Trpm4^+/+^* mice, *P*<0.05, n = 8 and 13 sections from *Trpm4^+/+^* and *Trpm4^-/-^* mice, respectively, Fig. 1F). At the ventricular level, the decrease in cell size and the corresponding increase in cell density suggest that cellular hypertrophy is not responsible for the increase in LVM. These results prompted us to hypothesize that there was an increase in the number of cardiomyocytes in *Trpm4^-/-^* mice.

### LV hypertrophy could be due to hyperplasia during proliferative stages

**Figure 2:**
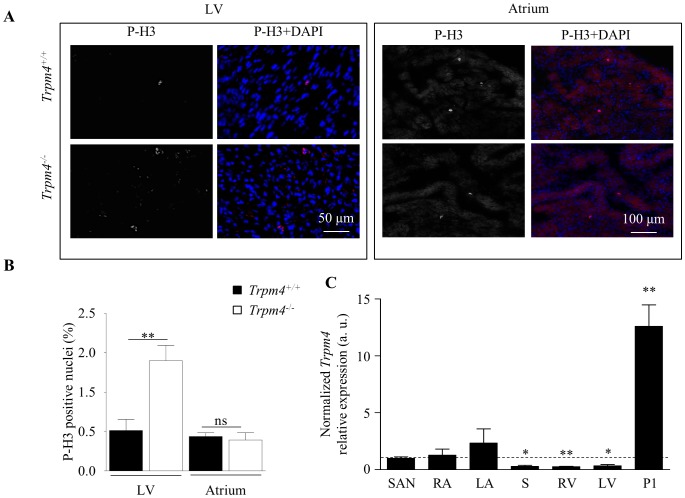
Hyperplasia during cardiomyogenesis in Trpm4^-/-^ neonatal mice. (**A**) Immunofluorescence labeling for phospho-histone H3 (P-H3, red) and counterstaining with DAPI (blue) in ventricle sections one day after birth (P1), viewed under a 40X objective in the left panel. Immunofluorescence labeling for P-H3 (red) and counterstaining with DAPI (blue) in atrial sections one day after birth (P1), viewed under a 20X objective in right panel. (**B**) Histograms represent mean number of P-H3-positive nuclei for each atrial or ventricular section (3 mice per group for the atria and 7 *Trpm4^+/+^* and 8 *Trpm4^-/-^* mice for ventricle). **: *P*<0.01, ns: non-significant. (**C**) Quantitative reverse transcription-polymerase chain reaction assessment of mRNA from sino-atrial node (SAN), right atria (RA), left atria (LA), septum (S), right ventricular tissue (RV) and left ventricular tissue (LV), presented relative to the expression of housekeeping gene in arbitrary units (a.u.) (*Gapdh* in adult tissue or*14S* ribosomal in postnatal day 1 (P1) tissue). Every relative expression was then normalized to the *Trpm4* SAN expression. *statistical analysis comparison with SAN, *: *P*<0.05, **: *P*<0.01.

Cardiomyocytes actively proliferate during embryonic, fetal, and neonatal stages [24], [25]. The increase of cell density in *Trpm4^-/-^* mice could be explained by an increase of cell proliferation at these stages. We thus assessed the proliferative state of myocytes in neonates by immunofluorescence labeling with the mitosis marker phospho-histone H3 (P-H3), a mitosis marker. P-H3 labeling was increased 3-fold in ventricular cryosections from *Trpm4^-/-^* mice (n = 8 sections) (*vs. Trpm4^+/+^* mice, n = 7 sections) one day after birth (postnatal day 1, P1) whereas no difference was observed in the atria (n = 3 per groups, Fig. 2A-B). Using quantitative RT-PCR, we determined that TRPM4 mRNA levels were more than 10-fold higher in the heart of wild-type neonate animals than in other regions of the adult heart (sinus atrial node, ventricles, septum and atria) (Fig. 2C). These data suggested that TRPM4 is highly expressed in the neonatal stage, when hyperplasia is detected. It is an appealing hypothesis to imagine that the TRPM4 channels could be involved in the regulation of cardiomyocytes proliferation during heart development. Further experiments are warranted to validate this possibility.

### *Trpm4^-/-^* mice exhibit multilevel conduction blocks and bursts of repetitive ectopic atrial activity

**Figure 3:**
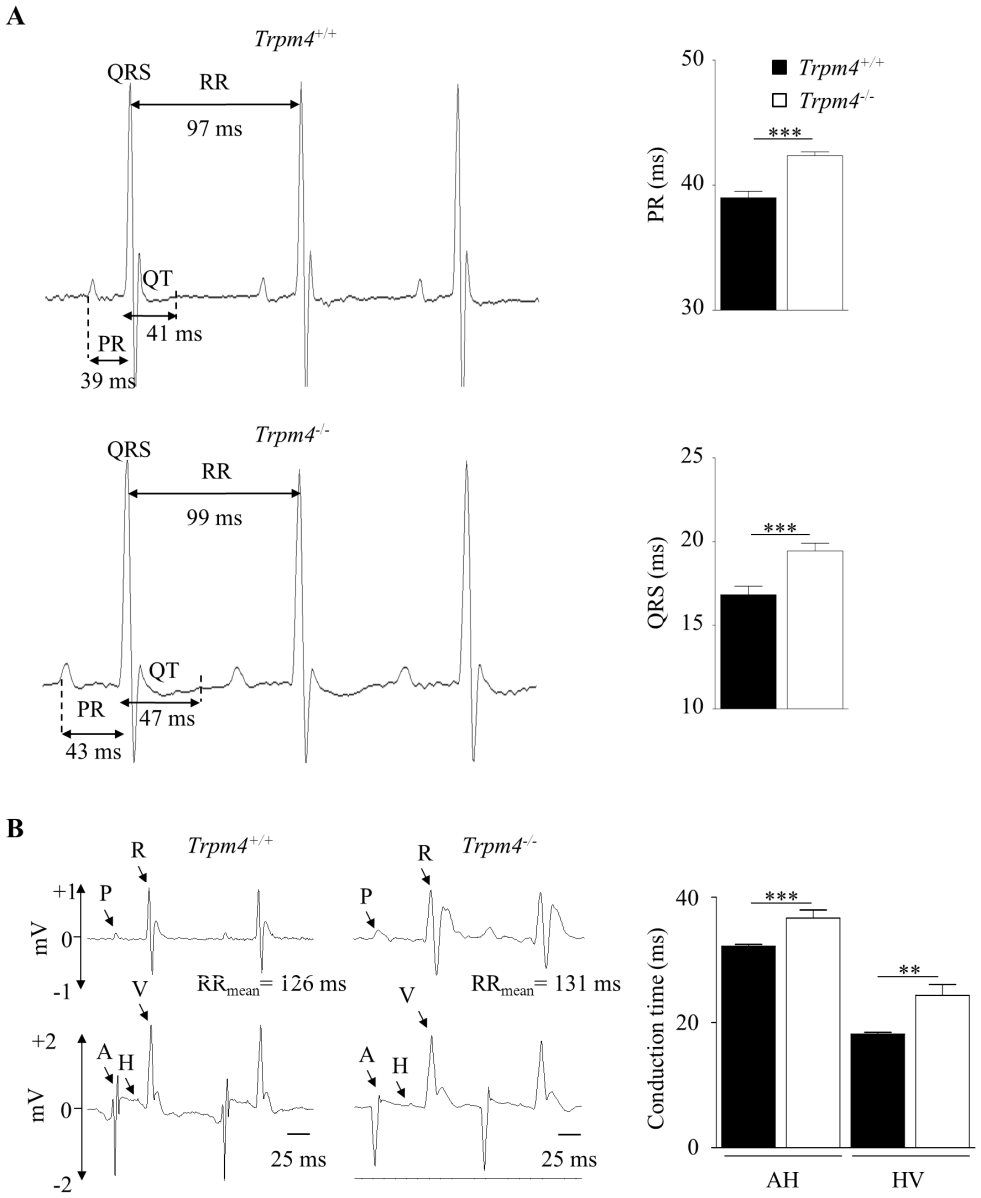
Electrocardiograms (ECGs) and intracardiac conduction analyses in Trpm4^+/+^ and Trpm4^-/-^ mice. 24h ECGs were acquired by telemetry in conscious mice, and 12h nocturnal periods were analyzed. (**A**) Typical ECGs, PR and QRS durations. Data are expressed as the mean of 13 *Trpm4^+/+^* and 18 *Trpm4^-/-^* mice. (**B**) Intracardiac conduction analysis. Atrial, His bundle and ventricular electrical activities were recorded. Top: surface ECG; Bottom: intracardiac electrical activity. P: P wave; R: R wave; A: atrium; H: His bundle; V: ventricle. AH (suprahisian) and HV (infrahisian) intervals. Data are expressed as the mean ± S.E.M. of 6 *Trpm4^+/+^* and 6 *Trpm4^-/-^* mice. **: *P*<0.01, ***: *P*<0.001.

We next investigated the consequences of *Trpm4* gene deletion in atria and conduction system on cardiac electrical activity by measuring surface electrocardiograms. Surface ECGs were recorded in freely moving mice at 12 weeks of age (Fig. 3A). The heart rate (expressed as the RR interval) was similar in *Trpm4* ^-/-^ (99.9±1.6 ms, n = 18) and *Trpm4* ^+/+^ (97.5±0.9 ms, n = 13; *P* = 0.24) animals, as reported previously [12]. The lack of modification of the basal heart rate, as previously shown [13], suggests however that TRPM4 does not greatly contribute to basal pacemaker activity conversely to that reported in microelectrodes experiments performed on spontaneously beating isolated atria [15]. The heart rate variability (HRV), an indicator of autonomic nervous system regulation of cardiac function, was also similar in the two groups, as indicated by the mean standard deviation of normal-to-normal heart rate (SDNN) over 12 hours (9.9±0.5 ms in *Trpm4* ^-/-^ *vs.* 9.1±0.6 ms in *Trpm4* ^+/+^; *P* = 0.3). In contrast, electrical conduction in *Trpm4^-/-^* hearts was disturbed as shown by 1^st^ degree atrioventricular blocks (AVBs; *i.e*. an increase in the PR interval), and broadening of the QRS complex, illustrating bundle branch blocks in *Trpm4* ^-/-^when compared to *Trpm4^+/+^* mice (PR: 42.3±0.3 *vs.* 39±0.5 ms, *P*<0.001; QRS: 19.4±0.4 *vs.* 16.8±0.5 ms, *P*<0.001) (Fig. 3A). The QT interval was also prolonged in *Trpm4^-/-^* mice (47.4±0.7 *vs.* 41±0.7 ms in *Trpm4^-/-^* and *Trpm4^+/+^* mice, respectively; *P*<0.001). The corrected QT interval was calculated based on the Bazett' formula and was also increased in *Trpm4^-/-^* mice (42.1±0.8 *vs*. 47.3±0.6 ms for *Trpm4^+/+^* and *Trpm4^-/-^* mice, respectively, *P value*<0.0001)

The slowing of electrical propagation in *Trpm4* ^-/-^ mice was confirmed by intracardiac electrophysiological exploration (Fig. 3B). Both suprahisian and infrahisian conduction times were lengthened in *Trpm4* ^-/-^ compared to *Trpm4* ^+/+^mice (AH: 36.6±1.3 *vs.* 32.1±0.3 ms, *P*<0.01; HV: 24.3±1.8 *vs.* 18.1±0.3 ms; *P*<0.01). Thus, *Trpm4* ^-/-^ mice exhibited slowed electrical conduction at all cardiac stages. In parallel, we investigated the expression of connexins (Cx) 30.2, 40, 43 and 45, proteins essential for electrical coupling between cardiac cells and involved in electrical conduction. The expression of connexins was similar in the right atrium and in the left ventricle from *Trpm4^-/-^* and *Trpm4^-/-^*, except for Cx30.2, a conduction-slowing connexin [26], which was increased in the right atrium (S2A Fig.). However, the protein amount of Connexin 30.2 (normalized to Calsequestrin 2 protein), assessed by western blot analysis, was not significantly different between *Trpm4^-/-^* and *Trpm4^-/-^* mice (S2B Fig.) (1.13±0.2 *vs.* 1.3±0.05 in a.u. in atrial lysates from *Trpm4^+/+^ and Trpm4^-/-^* mice, respectively, n = 3 for both groups *P* = 0.43). Surprisingly, Connexin 40 protein expression was significantly lower in *Trpm4^-/-^* atria when compared with *Trpm4^+/+^* atria (S2B Fig.) (1.19±0.15 *vs.* 0.72±0.1 a.u. in lysates from *Trpm4^+/+^ and Trpm4^-/-^* atria, respectively, n = 3, *P* = 0.017). This result suggests that the slowing conduction time, at least in atria, observed in both ECG and intracardiac analysis, could be due to the Cx40 protein expression modifications in line with other reports [27], [28].

**Figure 4:**
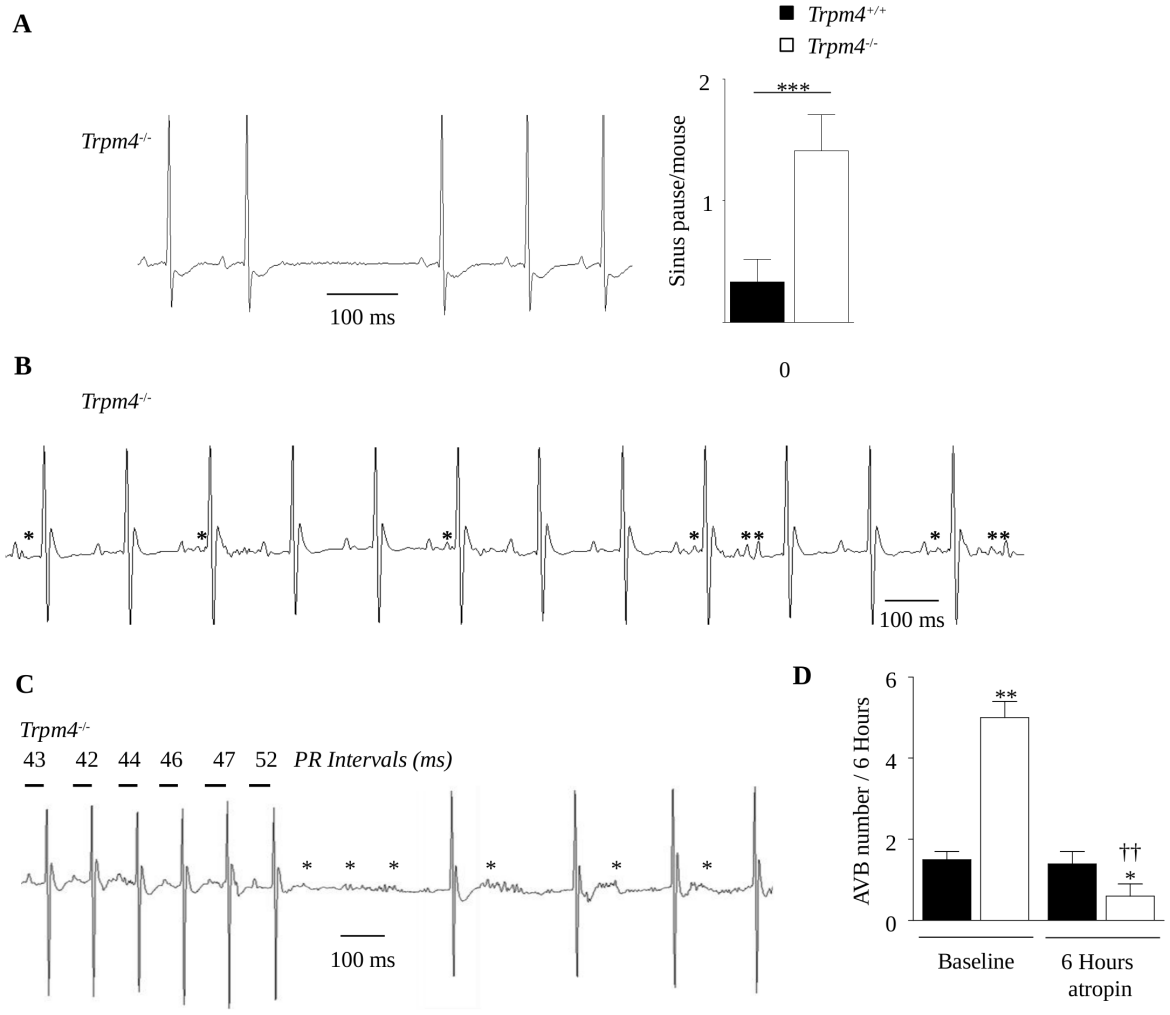
Abnormal electrical activity in Trpm4^-/-^ mice. Arrhythmic events were counted during 12h nocturnal periods according to the Lambeth convention (**A**) Typical sinoatrial node (SAN) pause. Histogramm is the mean number of sinus pauses in *Trpm4^-/-^ vs. Trpm4^+/+^* mice. (**B**) Representative trace of ectopic atrial activities in *Trpm4^-/-^* mice. Asterisks represent ectopic P waves. (**C**) ECG recorded in a *Trpm4^-/-^* mouse illustrating the lengthening of the PR interval for 4–6 beats before the appearance of the AVB. Asterisks represent P waves not followed by a QRS complex. (**D**) Number of AVBs in *Tpm4^+/+^*and *Trpm4^-/-^* mice before and during 6 hours of atropine infusion. Data are expressed as the mean ± S.E.M. of 13 *Trpm4^+/+^* and 18 *Trpm4^-/-^* mice (A–C) and the means ± S.E.M. of 5 *Trpm4^+/+^* and 5 *Trpm4^-/-^* mice (D); ns: no significant difference; *: *P*<0.05, **: *P*<0.01, (A–C) and *: *Tpm4^+/+^ vs. Trpm4^-/-^*, *: *P*<0.05, **: *P*<0.01. † *vs.* baseline of each group (Wilcoxon matched pairs test), ††: *P*<0.01 (D).

Moreover, *Trpm4* ^-/-^ mice exhibited punctual absences of the P wave corresponding to sinus arrests or sinoatrial blocks (for a 12-hour period: 1.41±0.3, n = 18 *vs.* 0.33±0.2 pause/mouse, n = 13 in *Trpm4* ^+/+^ mice; *P*<0.05) (Fig. 4A). *Trpm4* ^-/-^ mice also displayed more repetitive ectopic atrial activity compared to *Trpm4^-/-^* mice (Fig. 4B). In association with electrical conduction disorders, *Trpm4* ^-/-^ mice exhibited a higher incidence of Mobitz type-I 2^nd^ degree AVBs (or Luciani-WenckebachAVBs) compared to*Trpm4* ^+/+^ animals (S3A Fig.). A progressive prolongation of the few PR intervals occurred exclusively and immediately prior to the blocks (Fig. 4C) [29]. The SDNN associated with the 6 beats preceding the AVBs was markedly increased (12.5±0.5 ms, n = 18 *vs.* 9.9±0.5 ms in *Trpm4* ^+/+^, n = 13; *P*<0.01). The progressive PR lengthening (S3B Fig.), which was not observed in *Trpm4* ^+/+^ animals, appeared concomitantly with an increase in the short-term HRV parameter RMSSD (root mean square of the difference between adjacent normal RR intervals) (S3C Fig.) suggesting that progressive PR lengthening leading to AVBs was due to paroxysmal parasympathetic overdrive. Altogether, these data suggest that the absence of TRPM4 slows electrical conduction, favoring the generation of arrhythmias in part through the dysregulation of the cardiac autonomic nervous system. To further examine this hypothesis, we recorded ECGs during 6 hours of infusion with atropine, a parasympatholytic agent. During atropine infusion, the RR interval was unchanged probably due to weak vagal tone in mice [30]–[32]. As expected, atropine decreased the occurrence of Luciani-Wenckebach AVBs in *Trpm4^-/-^* mice (from 5.0±0.4 to 0.6±0.3 AVB/6 hours, *P*<0.01, n = 3 mice per group), whereas the number of AVBs in wild-type mice was unchanged (1.5±0.2 *vs.* 1.4±0.3 AVB/6hours, ns) (see Fig. 4D and S3D Fig.). These results reinforced the hypothesis that the Luciani-Wenckebach AVBs observed in *Trpm4^-/-^* mice originated from vagal overdrive. In contrast, atropine had no effect on the mean PR duration in *Trpm4^+/+^* or *Trpm4^-/-^* mice, suggesting that 1^st^degree AVBs were not mediated by chronic parasympathetic over activity, but rather by structural and/or ionic changes.

### *Trpm4^-/-^* mice exhibit shorter APs in atrial cells but normal APs in the left ventricular cardiomyocytes

**Figure 5:**
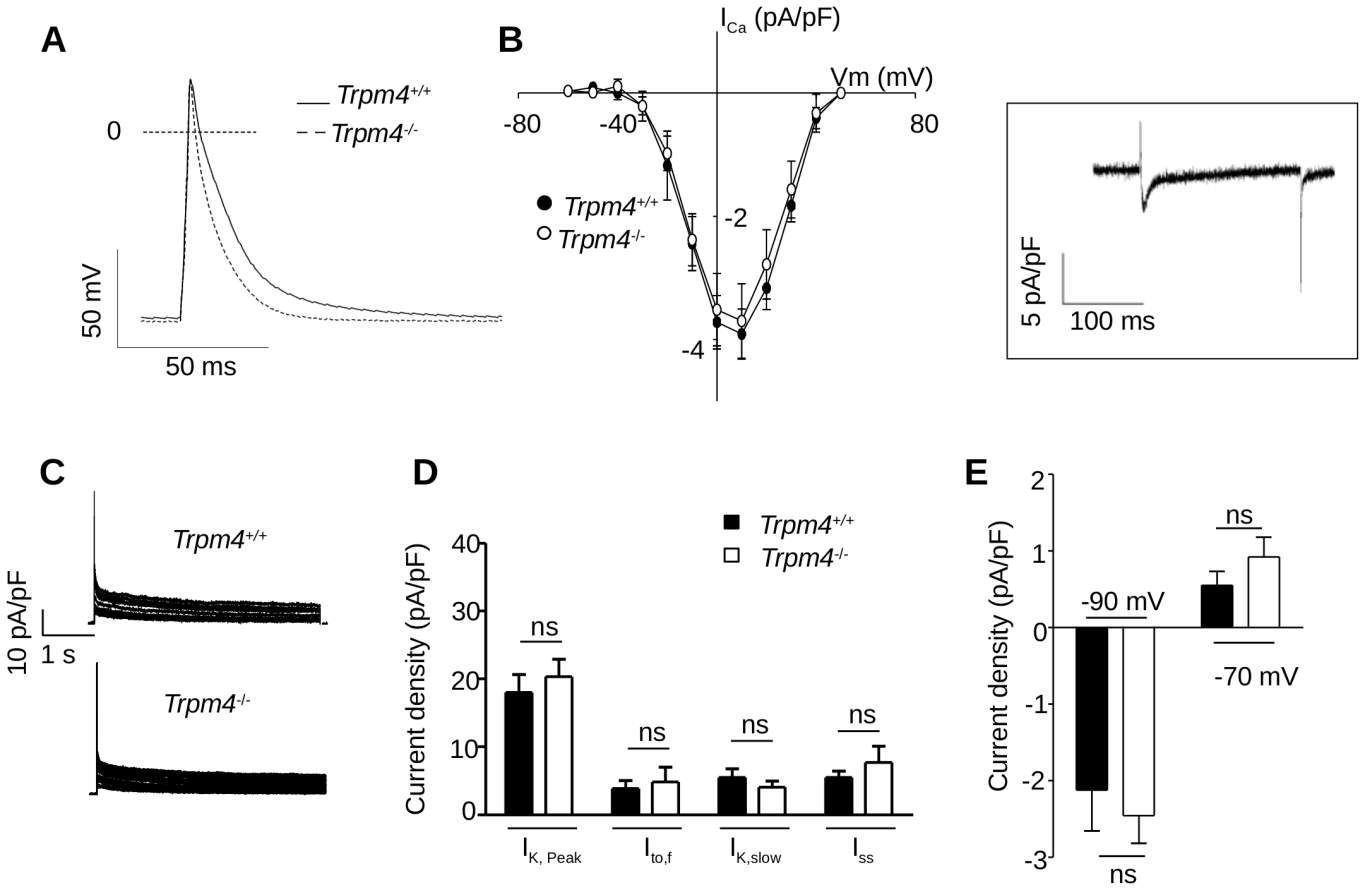
Direct contribution of the TRPM4 channel to AP waveform in isolated atrial cardiomyocytes. (**A**) Mean AP waveforms recorded from Trpm4^+/+^ and Trpm4^-/-^ atrial cells. (**B**) Density of I_Ca,L_ plotted as a function of voltage in Trpm4^+/+^ and Trpm4^-/-^ atrial myocytes. Inset: representative I_Ca,L_ from a Trpm4^-/-^ atrial myocyte at 0 mV. (**C**) Representative outward voltage-gated K^+^ current traces recorded on freshly isolated cardiomyocytes from Trpm4^+/+^ (upper trace) and Trpm4^-/-^ (lower trace) mice. (**D**) Current densities of I_K,peak_, I_to,f_, I_K,slow_ and I_SS_ (at +20 mV) in atrial myocytes isolated from Trpm4^+/+^ and Trpm4^-/-^mice. (**E**) I_K1_ current densities (at −90 and −70 mV) measured from Trpm4^+/+^ and Trpm4^-/-^ atrial myocytes. Data are expressed as the mean ± S.E.M. of at least 6 atrial cells from *Trpm4^+/+^* and *Trpm4^-/-^* mice; ns: no significant difference.

**Figure 6:**
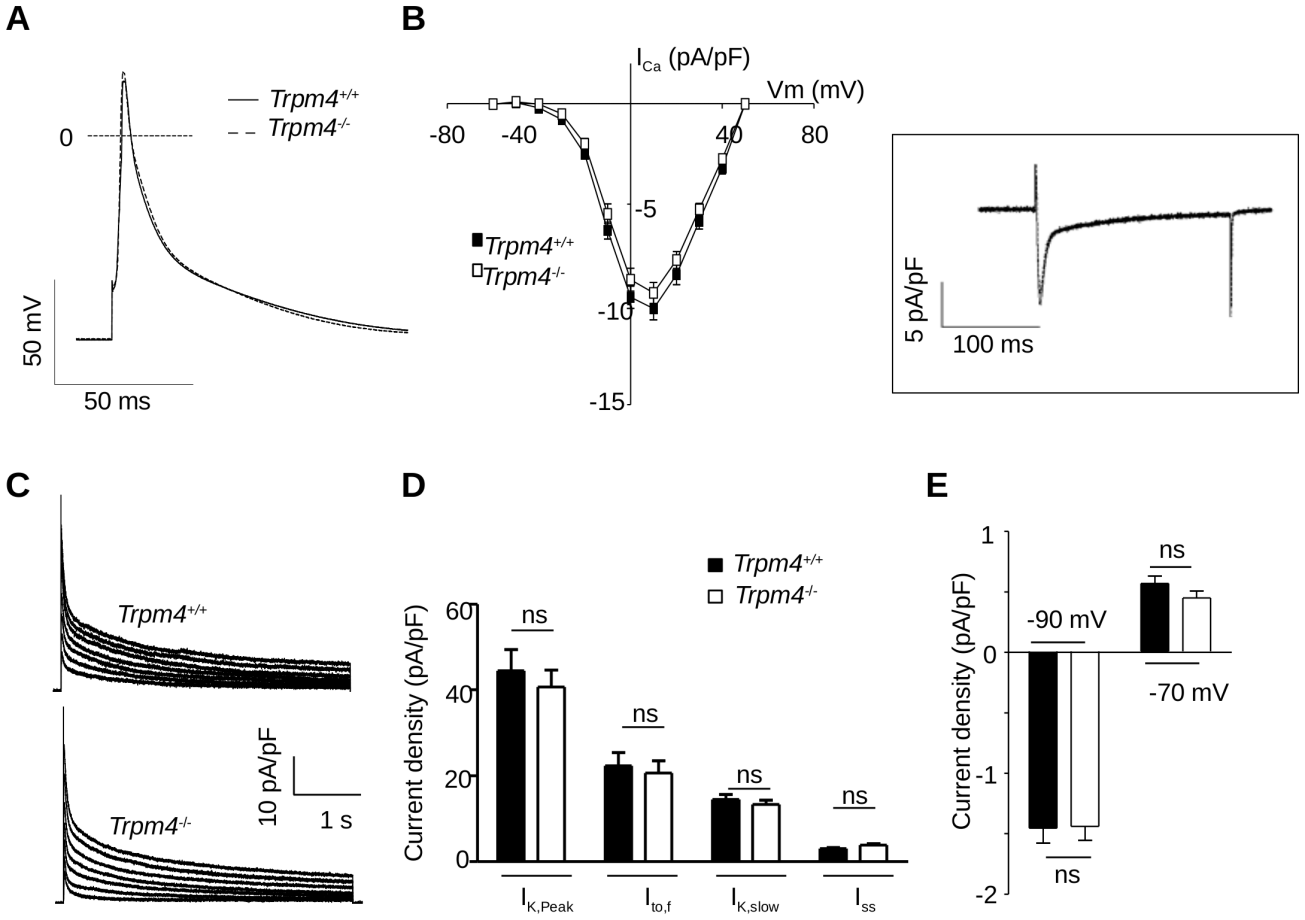
No significant role of the TRPM4 channel to AP waveform in isolated ventricular cardiomyocytes. (**A**) Mean AP waveforms recorded from *Trpm4^+/+^* and *Trpm4^-/-^* LV myocytes. (**B**) Density of I_Ca,L_ plotted as a function of voltage in *Trpm4^+/+^* and *Trpm4^-/-^* LV myocytes. *Inset:* representative I_Ca,L_ from a *Trpm4^-/-^* LV myocyte at 0 mV. (**C**) Representative outward voltage-gated K^+^ current traces recorded on freshly isolated LV cardiomyocytes from *Trpm4^+/+^* (upper trace) and *Trpm4^-/-^* (lower trace) mice. (**D**) Current densities of I_K,peak_, I_to,f_, I_K,slow_ and I_SS_ (at +20 mV) in atrial myocytes isolated from *Trpm4^+/+^* and *Trpm4^-/-^*. (**E**) I_K1_ current densities (at −90 and −70 mV) measured from *Trpm4^+/+^* and *Trpm4^-/-^* LV myocytes. Data are expressed as the mean ± S.E.M. of at least 14 ventricular cells from *Trpm4^+/+^* and *Trpm4^-/-^*mice; ns: no significant difference.

To assess if the absence of TRPM4 directly affected ionic homeostasis, we recorded APs of freshly isolated atrial and ventricular cardiomyocytes. In atrial cells, the AP recorded using the whole-cell patch clamp technique was shorter in *Trpm4^-/-^* mice than in *Trpm4* ^+/+^ animals in line with recent results using microelectrodes and associated with pharmacological assessments [14].In particular, the APD_50_ and APD_90_ were decreased (Fig. 5A and Table 6). In contrast, neither the resting membrane potential nor the AP upstroke velocity (dV/dt) was modified. As AP shortening may reflect alteration(s) or remodeling of other ionic currents, we investigated the main K^+^ and I_Ca,L_ currents involved in the AP repolarizing phase. Analysis of the current-to-voltage relationship of peak I_Ca,L_(Fig. 5B) and steady-state availability for opening (data not shown) showed no difference in the density and voltage-dependent properties of this current between *Trpm4^-/-^* and *Trpm4^+/+^* cells. The decay kinetics, which also contributes to AP repolarization [33], were similar as well in both groups (τ_fast_ was 7.33±0.7 *vs.* 9.74±2 ms, *P* = 0.29; and τ_slow_ was 157±24 *vs.* 109±21 ms, *P* = 0.14; *Trpm4^-/-^* (n = 9) *vs. Trpm4^+/+^* (n = 12)). The different repolarizing voltage-gated outward K^+^ currents, I_K,peak_, I_to_, I_K,slow_ and I_ss_, measured as defined previously [34] as well as the inward rectifying K^+^ current I_K1_ (Fig. 5C–E) were unchanged. In contrast to atrial cells and to a recent study [13], the AP waveform in ventricular cardiomyocytes was similar in *Trpm4^-/-^* and *Trpm4* ^+/+^ mice (Fig. 6 and Table 6), in line with poor expression of the TRPM4 protein in adult LV cells (Fig. 2C and [8], [10], [11]). Consistently, both I_Ca,L_ (amplitude, voltage-dependent properties and decay kinetics) and K^+^ currents were similar in *Trpm4^-/-^* and *Trpm4^+/+^* mice (Fig. 6 B-E). We concluded that TRPM4, in basal conditions (absence of disease), contributes substantially in shaping the AP in atrial cells but not in single ventricular cells.

**Table 6:** AP characteristics from freshly isolated atrial and ventricular cardiomyocytes.

Parameters	*Trpm4^+/+^*	*Trpm4^-/-^*	*P value*
**Atrial RMP (mV)**	−90.5±3.3	−96±2.5	ns
**Atrial AP amplitude (mV)**	130.4±5.1	136.5±4.8	ns
**Atrial APD_20_ (msec)**	2.4±0.4	1.4±0.1	ns
**Atrial APD_50_ (msec)**	9.9±1.4	5.9±0.9	*
**Atrial APD_90_ (msec)**	35.7±5.2	19.4±2.6	*
**Atrial dV/dt (volt/sec)**	111.5±15.9	116.7±13.5	ns
**Ventricular RMP (mV)**	−82.4±1.6	−81.9±1.4	ns
**Ventricular AP amplitude (mV)**	127.4±2.2	131.6±1.8	ns
**Ventricular APD_20_ (msec)**	0.93±0.1	1.1±0.1	ns
**Ventricular APD_50_ (msec)**	3.65±0.7	4.79±0.6	ns
**Ventricular APD_90_ (msec)**	37.32±4.5	38.3±4.6	ns

Values are mean±SEM, n =  12 and 11 atrial cells and 13 and 31 ventricular cells from *Trpm4^+/+^* and *Trpm4^-/-^,* respectively. RMP: resting membrane potential, AP: action potential, APD_20,_ APD_50_ and APD_90_: Action potential duration at 20, 50 and 90% of repolarization time, dV/dt: rate of rise of AP.*, *P*<0.05 ns, non significant.

## Discussion

In this study, we showed that deletion of the *Trpm4* gene in mice alters the cardiac phenotype with morphological and electrical changes. *Trpm4^-/-^* mice exhibited cardiac hypertrophy, higher cellular density and smaller LV cardiomyocytes size at the age of 12 weeks. LV cardiomyocytes hyperplasia at birth suggested that *Trpm4* may act as a negative regulator of myocytes proliferation during prenatal development. The *Trpm4* ^-/-^ mice also exhibited electrical disorders, including multilevel conduction delays and blocks as well as paroxysmal runs of repetitive ectopic atrial beats, and shorter atrial AP that likely to favor ectopic activity.

*Trpm4^-/-^* mice exhibited moderate cardiac hypertrophy at 6 months of age (see also [12]), as well as ventricular dilation. The increase in both wall thickness and chamber size was consistent with a compensatory adaptation of heart proportions and function. The eccentric hypertrophic phenotype is usually associated with pressure overload, volume overload and contractile dysfunction. In particular, increased cardiac dimensions and LV contractility (moderate supranormal LV function) have been connected with systemic hypertension [35], [36]. Increased blood pressure arising from elevated plasma epinephrine levels has been shown in *Trpm4^-/-^* mice and may promote the development of hypertrophy overtime [12]. In the absence of typical hallmarks of hypertrophy such as fibrosis, cardiomyocytes hypertrophy (LV myocytes were actually smaller) and electrophysiological remodeling (no change in cellular AP), our findings advocated for the involvement of hyperplasia in the cardiac hypertrophy phenotype of *Trpm4^-/-^* mice [37]. Recently, a very elegant study [38], using mice invalidated for the *Trpm7^-/-^*gene, described similar effects on the embryonic and adult cardiac phenotype. In particular, *Trpm7^-/-^* mice displayed decreased hyperplasia associated with increased adult cardiomyocytes size (increased cell capacitance). TRPM7 is a Ca^2+^-permeating channel [39] whereas TRPM4 is a non-selective cationic channel regulating Ca^2+^ overload [40]. Interestingly, two different phenotypes were developed in *Trpm7^-/-^*mice adulthood: one developing cardiac hypertrophy with heart blocks (50% penetrant), and the other with normal heart size and devoid of heart blocks. Of note, in both groups, the *Trpm4* transcript was decreased, suggesting a potential link between TRPM7 and TRPM4 channels expression and/or regulation.

*Trpm4* may act as a negative regulator of hyperplasia and may also contribute to hypertrophy in adulthood [41]. The rapid switch from myocytes hyperplasia to hypertrophy occurs during early postnatal development (*i.e.* between P3 and P4 in mice), and is the major physiological mechanism underlying the increase in total myocytes mass during the postnatal period [42]. It is also a relevant mechanism in various pathological models in which exaggerated hyperplasia, resulting from the cytokinesis of differentiated cardiomyocytes, contributes to hypertrophy [37], [43]. Cardiomyocytes hyperplasia and proliferation have been described in a lethal neonatal familial form of dilated mitogenic cardiomyopathy [44]. Hyperplasia was also shown to promote eccentric hypertrophy in response to abnormal LV diastolic myocytes stress in anemia-induced cardiac hypertrophy in the rat [45]. The mechanisms underlying these changes are currently unclear. TRPM4 may be involved in Ca^2+^-mediated regulation of myocytes proliferation in the developing ventricle. Another hypothesis could be the consequences of increased catecholamine levels, shown in*Trpm4^-/-^* mice [12]. An involvement of β-adrenergic stimulation to neonate cardiomyocytes proliferation has been reported [46]. This latter hypothesis is attractive as the differential expression of adrenoreceptors in the atria and ventricles could explain the difference in hyperplasia between the two tissues [47], [48].

Another major finding of our study was the occurrence of multilevel conduction disorders in *Trpm4^-/-^*mice, suggesting that the TRPM4 channel plays a role in conduction both in the suprahisian and infrahisian territories as previously hypothesized [8], [18], [49]. *Trpm4^-/-^* mice exhibited constitutive PR and QRS lengthening as shown by surface ECGs, as well as the prolongation of both AH and HV intervals, evidenced by intracardiac exploration. Several mechanisms could mediate this overall slowing of electrical conduction. Tissue alterations, including an increase in cardiac mass and structural abnormalities such as fibrosis, are known to delay electrical propagation. Changes in the parasympathetic system may also well exert dromotropic changes. Finally, modifications of cellular electrophysiological properties frequently reduce conduction velocity via membrane hyperpolarization, a decreased fast depolarizing I_Na_, or the alteration of cell-cell communication through altered gap junction activity.

At the ventricular level, we and others, have found only weak expression of TRPM4 [8], [10], [11]. However, in conditions leading to cardiomyocytes hypertrophy either *in vivo* or *in vitro*, TRPM4 channel expression and function is likely to increase (concomitantly to fetal gene re-expression) [9], [11], [50], suggesting a role for TRPM4 in cellular hypertrophy [51]. Consistently, we found a high level of TRPM4 expression in neonatal ventricular cardiomyocytes in line with the presence of a NSC_Ca_ current sharing all of the properties of the TRPM4 current [9]. In the adult, the absence of fibrosis, altered connexins expression and AP modifications in the *Trpm4^-/-^* mice reinforces the concept that increased LVM due to hyperplasia was responsible for the conduction delay. An increase in LVM due to the higher density of cardiomyocytes could also contribute to the longer QRS interval [52]. The lack of involvement of fibrosis in this reduced conduction velocity is also confirmed by the absence of a fragmented QRS in surface ECGs in *Trpm4^-/-^* animals. It has been previously shown that cardiac myocytes proliferation may induce heart block [53]. We cannot, however, exclude modifications in the architecture or structure of the conductive tissue. Our results regarding the lack of AP waveform difference on ventricular cardiomyocytes between *Trpm4^+/+^*and *Trpm4^-/-^*mice are different with those obtained previously by Mathar et al. (2014). One possible explanation for this difference could be method in which AP measurements were recorded: Mathar *et al*. performed microelectrode AP measurements in tissue strips whereas we performed isolated cellular AP recordings. These differences in experimental conditions (isolated *vs.* tissue strip) do not allow for direct comparisons. As well, the background of the *Trpm4^-/-^* mouse was derived from the 129/SvJ strain and ours from the C57bl/6J strain. There is, more and more evidence that strain differences alter cardiac phenotype and regulation such as ß-adrenergic response [54]–[56]. These differences in experimental conditions and strain indicate that no clear evaluation can be made regarding the involvement of TRPM4 channels in wild-type ventricular cardiomyocytes electrical activity. Further attention is warranted to identify the source(s) of this discrepancy.

Numerous other studies have failed to detect functional TRPM4 current in ventricles [9]–[11] by inside-out patch-clamp technique. In addition, 9-Phenanthrol had no effect on ventricular AP waveform by microelectrode measurement while it decreased atrial APD in the same study [14].

Finally, in the majority of studies, only weak TRPM4 channel expression has been detectable in wild type mouse, rat, and human ventricles [8], [11], [57]. Conversely, Mathar and colleagues state that he presence of the TRPM4 protein expression in ventricle was demonstrated in their previous work [12] though we have not found the evidence supporting their finding.

Only two studies have shown an effect of the TRPM4 inhibitor 9-Phenanthrol in ventricles. These works investigated 9-Phenanthrol in hypoxia-reoxygenation and ischemia-reperfusion conditions [15], [17]. However, these are two pathological models in which it cannot be excluded that such conditions could affect (as in hypertrophy) either TRPM4 expression or function.

At the atrial level, in which TRPM4 is normally expressed, we observed 1^st^ degree AVBs (*i.e*. an increase in the PR interval) in *Trpm4^-/-^* mice. These conduction delays were unrelated to parasympathetic overactivity, increased atrial myocellular density or increased fibrosis. However, our finding that the Cx40 protein level was decreased in *Trpm4^-/-^* atria is in line with the PR interval increase. Cx40 protein is one of the major Cxs involved in AV conduction and Cx40-deficient mice display longer PR intervals associated with AH (and also HV) lengthening [27], [28]. In the atria, AP recordings demonstrate that the TRPM4 channel is involved in the AP duration. We have demonstrated that the main voltage-gated currents (I_Ca,L_, K_v_ and I_K1_), involved during repolarization, were similar in *Trpm4^-/-^* and *Trpm4^+/+^* mice, consistent with the pharmacological reduction of atrial AP, recorded with microelectrode in intact tissue, by the TRPM4 blocker 9-phenantrol [14]. TRPM4 is a Ca^2+^-activated non-selective cationic channel permeable to Na^+^ and K^+^ ions, but not Ca^2+^. However, TRPM4 senses intracellular Ca^2+^ following AP activation, which favors TRPM4 opening, generating an inward current in the negative range of voltages corresponding to AP repolarization. However, the dV/dt was unchanged in our study, suggesting that I_Na_ is not significantly altered. The resting membrane potential was also similar in *Trpm4^-/-^* and *Trpm4^+/+^* mice, suggesting that TRPM4 does not regulate electrical conduction through the modulation of cardiomyocyte membrane potential and, therefore, does not decrease the availability of Na^+^ channels capable of undergoing voltage-dependent opening. In contrast, ectopic atrial activity could be favored by this shortening of the AP duration and slowed conduction (probably due to the Cx40 protein decrease). As previously shown in humans, reduced Cx40 expression in atria and heterogeneity of its distribution may contribute to atrial fibrillation pathogenesis [58]. In addition, 2^nd^ degree type-I AVBs observed in *Trpm4^-/-^* mice in our study, which were abolished by atropine infusion in our study, seem to be related to paroxysmal parasympathetic overdrive. TRPM4 deletion leads to paroxysmal runs of repetitive ectopic atrial beats as well as shorter APD in atrial tissue, which could increase vulnerability to atrial tachyarrhythmia by favoring both the trigger and the re-entry phenomena. The implication of TRPM4 in the liberation of acetylcholine in autonomic ganglia [12] and the presence of TRPM4 in complex neurons of the brainstem are in line with our observations and its possible role in the autonomic regulation of cardiac function [59]. However, we cannot exclude that invalidation of the TRPM4 channel can lead to remodeling of ANS.

Overall, our results further support the idea that TRPM4 is a critical regulator of electrical conduction. The complexity of this regulation is evident in fact that both a gain [8] and a loss of function can lead to similar electrical disorders [18].

## Conclusion

In conclusion, TRPM4 is involved in the determination of heart size, potentially by negatively regulating hyperplasia. It also acts as a regulator of cardiac electrical conduction at the sinoatrial, atrioventricular, and intraventricular levels, and is directly involved in shaping the AP waveform of atrial myocytes. The *Trpm4^-/-^* mouse model may therefore represent a promising experimental model for the molecular dissection of the multiple and complex effects of TRPM4 on cardiac function.

## Supporting Information

S1 Fig **Trpm4^-/-^ mice develop eccentric hypertrophy without increased fibrosis.** (A) Histogram representing the relative wall thickness (RWT) at 32 weeks-old of age. Data are expressed as the mean of 8 *Trpm4^+/+^* and 7 *Trpm4^-/-^* mice. (B) Representative Goldner's trichrome staining in heart sections. (C) Quantitative RT-PCR for the expression of *Collagen1* (*Coll1*) and *Collagen3* (*Coll3*) genes in the left ventricle (LV), presented relative to the expression of *Gapdh* in arbitrary units (a.u.). ns: no significant difference.
(TIF)

S2 Fig **Connexin mRNA and protein levels in atrial and ventricular tissue of Trpm4^-/-^ and Trpm4^+/+^ mice.** (A) Quantitative RT-PCR expression of *Connexin 40* (*Cx40*), *Connexin 43* (*Cx43*), *Connexin 45* (*Cx45*) and *Connexin 30.2* (*Cx30.2*) in the right atrium (RA) and left ventricle (LV) *of Trpm4^+/+^* and *Trpm4^-/-^* mice, presented relative to the expression of *Gapdh* in arbitrary units (a.u). Data are expressed as the mean of at least 4 RAs and LVs per group. (B) Relative amount of connexin 43, 40 and 30.2 proteins in whole LV lysates (upper panel) or atrial lysates (lower panel) were determined calculating the Cx/CSQ (Calsequestrine 2) ratio. Data are representative of three independent experiments (n = 3 per group). ns: no significant difference. *: *P*<0.05 ***: *P*<0.001.
(TIF)

S3 Fig **The absence of TRPM4 slows electrical conduction.** (A) Mean number of atrioventricular blocks (AVBs) in *Trpm4^-/-^ vs. Trpm4^+/+^* mice. (B) Increase in the PR interval, and (C) of the root mean square of the difference (RMSSD) between successive normal intervals (in ms), reflecting short-term variations in HR due to parasympathetic activity immediately prior to AVBs in *Trpm4^-/-^* mice (*vs. Trpm4^+/+^*). (D) SDNN variability during 6 hours atropin injection by osmotic pump on 5 *Tpm4^+/+^* mice and 5 *Trpm4^-/-^* mice. Data are expressed as the means of 13 *Trpm4^+/+^* and 18 *Trpm4^-/-^* mice (A-C) and the means of 5 *Trpm4^+/+^* and 5 *Trpm4^-/-^* mice (D); ns: no significant difference; *: *P*<0.05, **: *P*<0.01, ***: *P*<0.001 (A–C) and *: *Tpm4^+/+^ vs. Trpm4^-/^*, *: *P*<0.05, **: *P*<0.01. † *vs.* baseline of each group (Wilcoxon matched pairs test), † †: *P*<0.01 (D).
(TIF)

S1 Checklist **NC3Rs, The ARRIVE Guidelines Checklist.** The ARRIVE guidelines describes the number and specific characteristics of animals used (including species, strain, sex, and genetic background); details of housing and husbandry; and the experimental, statistical, and analytical methods (including details of methods used to reduce bias such as randomisation and blinding).
(DOC)

S1 Supporting Information **Supplementary Methods and Results.** This file contains the methods used for Supplementary Figures such as western-blot analysis and Goldner's trichrome staining. It also contains additional data obtained by echocardiograms.
(DOCX)

S1 Table **Left Ventricular functional parameters in 32 weeks-old Trpm4^+/+^ and Trpm4^-/-^ sedated mice.** Values are mean ± SEM. LV EDV: Left Ventricular End-Diastolic Volume; LV ESV: Left Ventricular End-Systolic Volume; LVEF: Left Ventricular Ejection Function; LV mass corrected: Left Ventricular mass corrected; LVFS: Left Ventricular Fractional Shortening; LVOT: Left Ventricular Outflow Tract; * *Trpm4^+/+^vs. Trpm4^-/-^*; †12 *vs.* 32 weeks-old mice. * or † *P*<0.05, ** or †† *P*<0.01, *** or ††† *P*<0.001.
(DOCX)

S2 Table **Left Ventricular basal characteristics in 32 weeks-old Trpm4^+/+^ and Trpm4^-/-^ mice.** Values are mean ± SEM. IVS, ED and IVS, ES: End-Diastolic and End-Systolic InterVentricular Septum thickness; LVEDD and LVESD: Left Ventricular End-Diastolic and End-Systolic Diameters; LVPW, ED and LVPW, ES: End-Diastolic and End-Systolic Left Ventricular Posterior Wall Thickness. ns, non significant,* *Trpm4^+/+^vs.Trpm4^-/^*; †12 *vs.* 32 weeks-old mice. * or † *P*<0.05, ** or †† *P*<0.01, *** or ††† *P*<0.001.
(DOCX)
