# Supplementary figures and images for: Trpm4 Gene Invalidation Leads to Cardiac Hypertrophy and Electrophysiological Alterations
Source: PLoS One. 2014 Dec 22;9(12):e115256. doi: 10.1371/journal.pone.0115256 (PMC4274076; doi:10.1371/journal.pone.0115256)

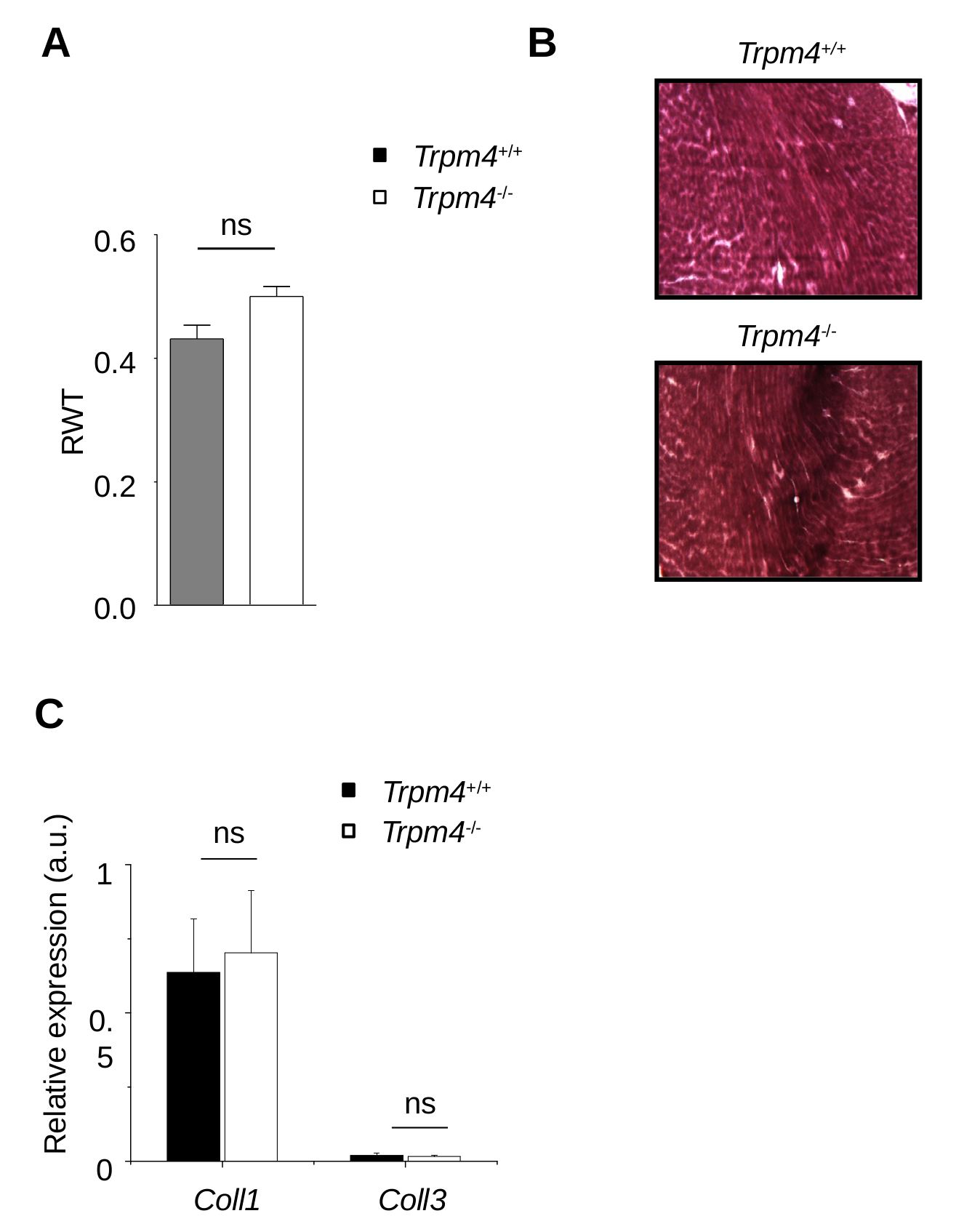

Supplement: S1 Fig — Trpm4-/- mice develop eccentric hypertrophy without increased fibrosis. (A) Histogram representing the relative wall thickness (RWT) at 32 weeks-old of age. Data are expressed as the mean of 8 Trpm4+/+ and 7 Trpm4-/- mice. (B) Representative Goldner's trichrome staining in heart sections. (C) Quantitative RT-PCR for the expression of Collagen1 (Coll1) and Collagen3 (Coll3) genes in the left ventricle (LV), presented relative to the expression of Gapdh in arbitrary units (a.u.). ns: no significant difference. (TIF) [file pone.0115256.s001.tif]

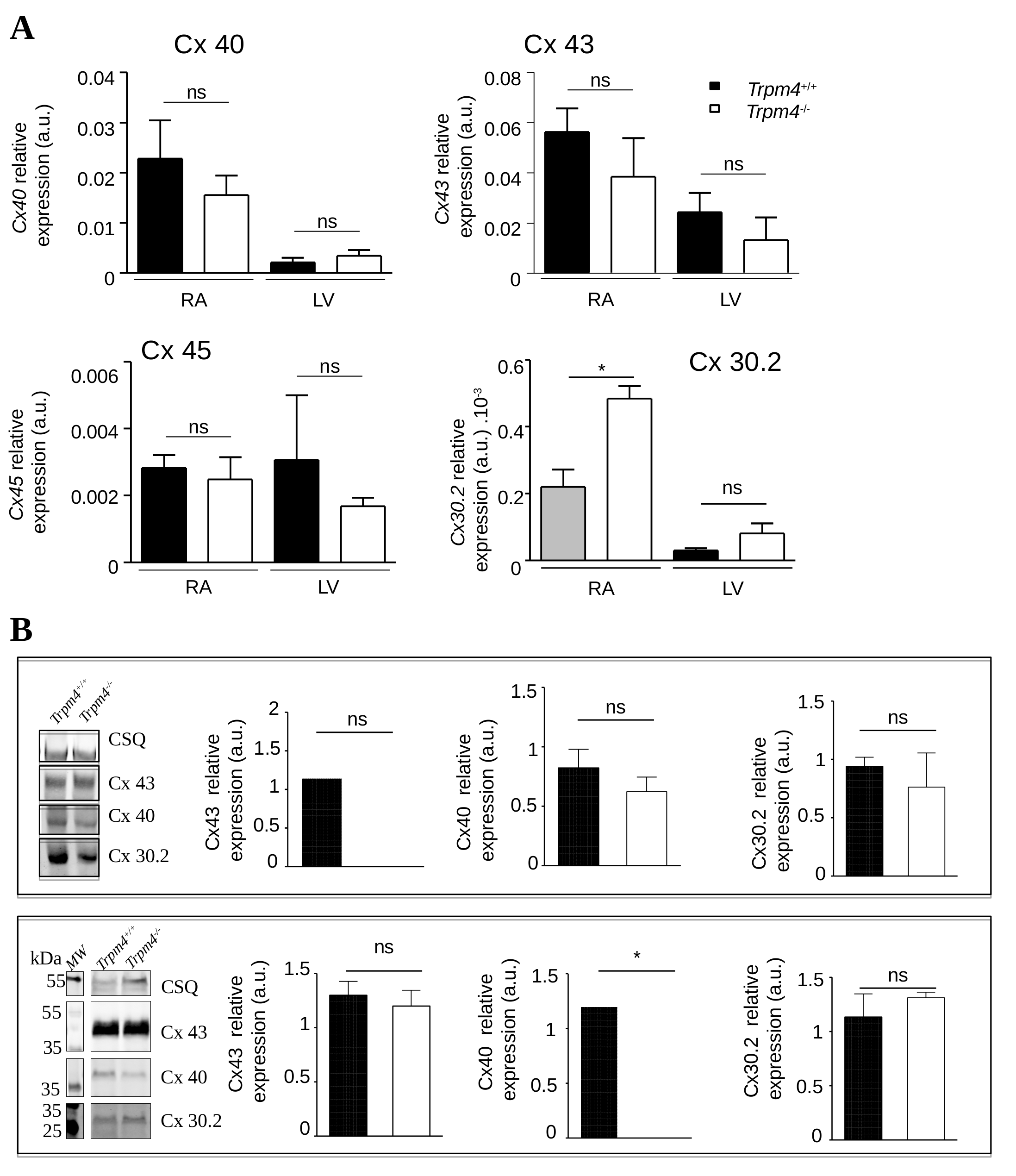

Supplement: S2 Fig — Connexin mRNA and protein levels in atrial and ventricular tissue of Trpm4-/- and Trpm4+/+ mice. (A) Quantitative RT-PCR expression of Connexin 40 (Cx40), Connexin 43 (Cx43), Connexin 45 (Cx45) and Connexin 30.2 (Cx30.2) in the right atrium (RA) and left ventricle (LV) of Trpm4+/+ and Trpm4-/- mice, presented relative to the expression of Gapdh in arbitrary units (a.u). Data are expressed as the mean of at least 4 RAs and LVs per group. (B) Relative amount of connexin 43, 40 and 30.2 proteins in whole LV lysates (upper panel) or atrial lysates (lower panel) were determined calculating the Cx/CSQ (Calsequestrine 2) ratio. Data are representative of three independent experiments (n = 3 per group). ns: no significant difference. *: P<0.05 ***: P<0.001. (TIF) [file pone.0115256.s002.tif]

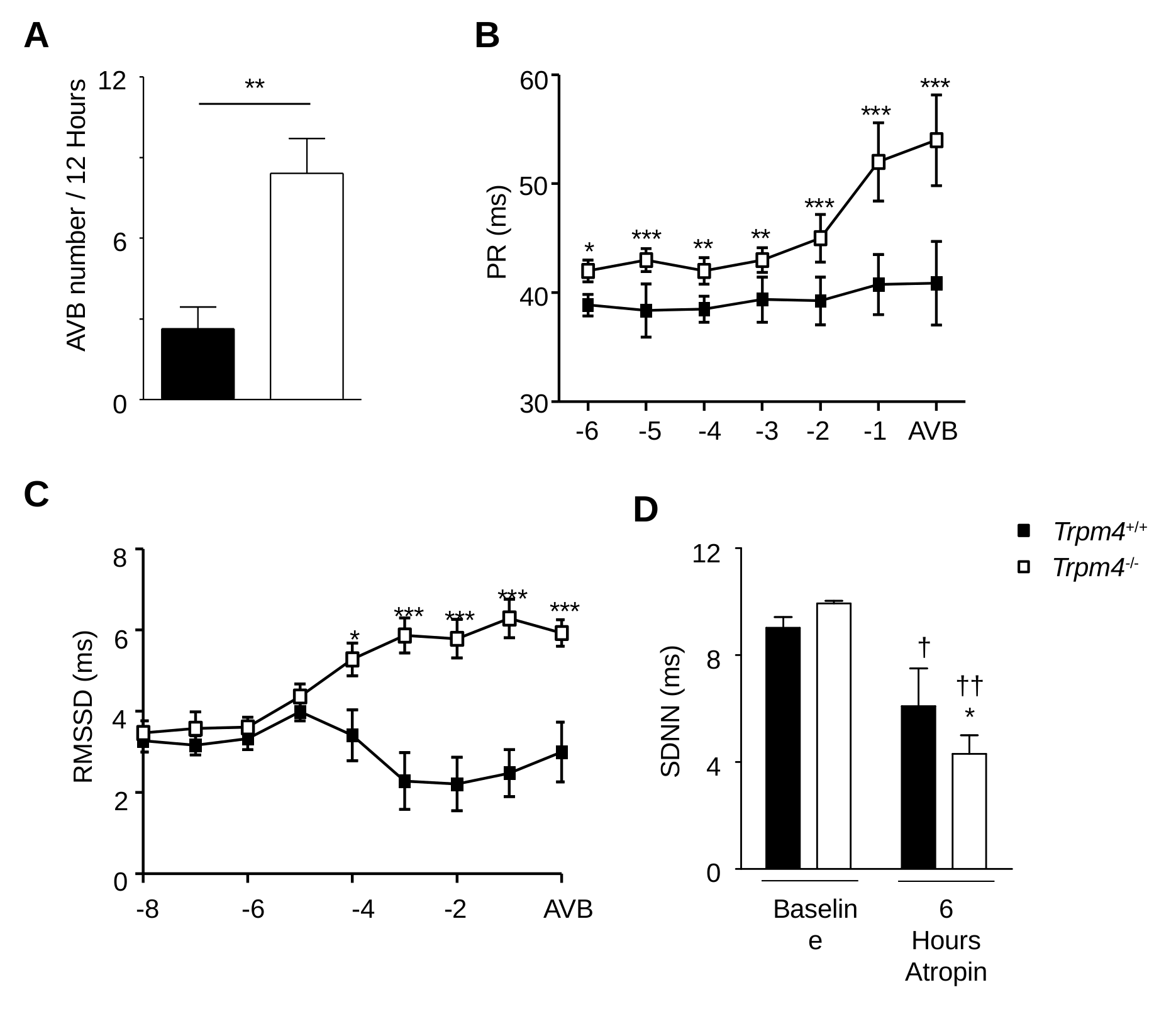

Supplement: S3 Fig — The absence of TRPM4 slows electrical conduction. (A) Mean number of atrioventricular blocks (AVBs) in Trpm4-/- vs. Trpm4+/+ mice. (B) Increase in the PR interval, and (C) of the root mean square of the difference (RMSSD) between successive normal intervals (in ms), reflecting short-term variations in HR due to parasympathetic activity immediately prior to AVBs in Trpm4-/- mice (vs. Trpm4+/+). (D) SDNN variability during 6 hours atropin injection by osmotic pump on 5 Tpm4+/+ mice and 5 Trpm4-/- mice. Data are expressed as the means of 13 Trpm4+/+ and 18 Trpm4-/- mice (A-C) and the means of 5 Trpm4+/+ and 5 Trpm4-/- mice (D); ns: no significant difference; *: P<0.05, **: P<0.01, ***: P<0.001 (A–C) and *: Tpm4+/+ vs. Trpm4-/, *: P<0.05, **: P<0.01. † vs. baseline of each group (Wilcoxon matched pairs test), † †: P<0.01 (D). (TIF) [file pone.0115256.s003.tif]
