# Supplementary material for: Trpm4 Gene Invalidation Leads to Cardiac Hypertrophy and Electrophysiological Alterations
Source: PLoS One. 2014 Dec 22;9(12):e115256. doi: 10.1371/journal.pone.0115256 (PMC4274076; doi:10.1371/journal.pone.0115256)
Supplement: S1 Supporting Information — Supplementary Methods and Results. This file contains the methods used for Supplementary Figures such as western-blot analysis and Goldner's trichrome staining. It also contains additional data obtained by echocardiograms. (DOCX) [file pone.0115256.s005.docx]

**SUPPORTING INFORMATION 1**

***Trpm4* gene invalidation leads to cardiac hypertrophy, multilevel conduction disorders and arrhythmias in mouse.**

Marie Demion^1,*^PhD, Jérôme Thireau^1,*^ PhD, Mélanie Gueffier^1^, Amanda Finan^1^ PhD, Ziad Khoueiry^1,3^, Cécile Cassan^1^PhD, Nicolas Serafini^2^ PhD, Franck Aimond^1^PhD, Mathieu Granier^1^ MD, Jean-Luc Pasquié^1,3^ PhD-MD, Pierre Launay^2^ PhD, Sylvain Richard^1^ PhD

^1^ INSERM U1046, Université Montpellier1, Université Montpellier2, Montpellier, France

^2^ Equipe Avenir, INSERM U1149, Université Paris 7, Paris, France

^3^ CHRU Montpellier, Service de Cardiologie, Montpellier, France

^*^ Equal contributions

**Supplemental Materials and Methods**

***Western-Blot analysis***

The expression of connexins (from whole LV or atrial lysates) was analyzed with 50µg of total protein. Proteins were separated by 12% SDS-PAGE and were transferred onto nitrocellulose membranes (0.2 μm, GE Healthcare, Brumath, France). Protein expression was assessed by immunoblot analysis with the primary antibodies anti-Connexin 30.2, anti-Connexin 40, anti-Connexin 43 (Life Technologies, St Aubin, France) or anti-Calsequestrin 2 (PA1-913, Fisher Scientific, Illkirch, France). Experiments were repeated a minimum of three times for each condition. All immunoblots were visualized and quantified using the Odyssey® infrared imaging system (LI-COR Biosciences, Lincoln, Nebraska, USA) coupled to infrared-labeled anti-goat, anti-mouse or anti-rabbit IgG secondary antibodies (1/30 000 dilution) (LI-COR Biosciences, Courtaboeuf, France). All primary and secondary antibodies were diluted in StartingBlock (TBS) blocking buffer.

**Goldner’s trichrome staining**

Fibrosis was assessed in ventricular cryosections (10 µm) using a Goldner's trichrome Diapath kit (Martinengo, Italy).

**Supplemental Results**

**Echocardiograms**

*Trpm4^-/-^* mice exhibited both septum and posterial wall thickness increase in end-diastolic and end-systolic period (as described in Table 1) at 12 as well as 32 weeks-old age.At 32 weeks, *Trpm4^-/-^* mice also displayed LV dilation assessed by the left ventricular diastolic diameter (4.36±0.10 mm*vs*.3.76±0.06 mm in *Trpm4^-/-^* and *Trpm4^+/+^* mice, respectively; *P*<0.001).

*Trpm4^-/-^*mice developed a left circumferential and symmetric hypertrophy. Actually, the inter-ventricular septum (IVS) and the left posterior ventricular posterior wall (LVPW) were significantly thicker. *Trpm4^+/+^* and *Trpm4^-/-^* mice exhibited different structural and functional characteristics (Table 2). Finally, *Trpm4^-/-^* mice, whatever the age, had an increased left ventricular mass (LVM corrected and normalized to BW was 4.1±0.6 mg/g *vs.* 3.06±0.1 mg/g in 12 weeks-old *Trpm4^-/-^* and *Trpm4^+/+^*mice, respectively; *P*<0.01 and 4.96±0.6 mg/g *vs.* 3.12±0.09 mg/g in *Trpm4^-/-^* and *Trpm4^+/+^*in 32 weeks-old mice, respectively; *P*<0.001).

Unexpectedly, these structural modifications were not associated with a decline of the left systolic and diastolic function but rather left ventricular contractile properties were preserved (Table 2). Consistently, the fractional shortening (FS %), which was similar at 12 weeks (35.2±3.0 % *vs.* 34.2±4.2% in *Trpm4^-/-^*and *Trpm4^+/+^*mice, respectively; no significant) was slightly increased at 32 weeks (36.3±2.0 % for *Trpm4^-/-^*mice *vs*. 29.3±1.8 % for *Trpm4^+/+^*mice; *P*=0.04), and the left ventricular ejection fraction (EF %) tended to increase at 32 weeks (65.7±2.7% *vs*. 56.3±2.6 %, in *Trpm4^-/-^*and *Trpm4^+/+^* mice, respectively; *P*=0.06 non significant). Altogether, these data clearly indicate that *Trpm4^-/-^*mice displayed preserved left ventricular systolic function.
