# Supplementary material for: Trpm4 Gene Invalidation Leads to Cardiac Hypertrophy and Electrophysiological Alterations
Source: PLoS One. 2014 Dec 22;9(12):e115256. doi: 10.1371/journal.pone.0115256 (PMC4274076; doi:10.1371/journal.pone.0115256)
Supplement: S1 Table — Left Ventricular functional parameters in 32 weeks-old Trpm4+/+ and Trpm4-/- sedated mice. Values are mean ± SEM. LV EDV: Left Ventricular End-Diastolic Volume; LV ESV: Left Ventricular End-Systolic Volume; LVEF: Left Ventricular Ejection Function; LV mass corrected: Left Ventricular mass corrected; LVFS: Left Ventricular Fractional Shortening; LVOT: Left Ventricular Outflow Tract; * Trpm4+/+vs. Trpm4-/-; †12 vs. 32 weeks-old mice. * or † P<0.05, ** or †† P<0.01, *** or ††† P<0.001. (DOCX) [file pone.0115256.s006.docx]

**Table S1**: **Left Ventricular functional parameters in 32 weeks-old *Trpm4^+/+^* and *Trpm4^-/-^* sedated mice.**

|  | *Trpm4^+/+^* |  | | *Trpm4^-/-^* |  | | *P value* |  |
| --- | --- | --- | --- | --- | --- | --- | --- | --- |
| Parameters | ***32 weeks (n=8)*** | | ***P value*** | ***32 weeks (n=7)*** | | ***P value*** | ***32 weeks*** |  |
| LV mass corrected (mg/g) | 3.12 ± 0.1 | | ns | 4.96 ± 0.6 | | ns | *** |  |
| LV EDV (µL) | 63.3 ± 3.1 | | ns | 83.9 ± 3.8 | | †† | ** |  |
| LV ESV (µL) | 33.0 ± 3.4 | | ns | 34.05 ± 3.4 | | ns | ns |  |
| Stroke volume corrected (µL/g) | 1.70 ± 0.15 | | ns | 1.68 ± 0.3 | | ns | ns |  |
| LVEF (%) | 56.31 ± 2.6 | | ns | 65.7 ± 2.7 | | ns | ns |  |
| LVFS (%) | 29.3 ± 1.7 | | ns | 36.3 ± 2.0 | | ns | * |  |
| LVOT (mm) | 1.25 ± 0.02 | | ns | 1.21 ± 0.1 | | † | ns |  |
| CO corrected ((mL/min)/g) | 0.62 ± 0.2 | | ns | 0.69 ± 0.2 | | ns | ns |  |

Values are mean ± SEM. LV EDV: Left Ventricular End-Diastolic Volume; LV ESV: Left Ventricular End-Systolic Volume; LVEF: Left Ventricular Ejection Function; LV mass corrected: Left Ventricular mass corrected; LVFS: Left Ventricular Fractional Shortening; LVOT: Left Ventricular Outflow Tract; * *Trpm4^+/+^vs. Trpm4^-/-^*; †12 *vs.* 32 weeks-old mice. * or † *P*<0.05, ** or †† *P*<0.01, *** or ††† *P*<0.001.
