# Supplementary material for: Trpm4 Gene Invalidation Leads to Cardiac Hypertrophy and Electrophysiological Alterations
Source: PLoS One. 2014 Dec 22;9(12):e115256. doi: 10.1371/journal.pone.0115256 (PMC4274076; doi:10.1371/journal.pone.0115256)
Supplement: S2 Table — Left Ventricular basal characteristics in 32 weeks-old Trpm4+/+ and Trpm4-/- mice. Values are mean ± SEM. IVS, ED and IVS, ES: End-Diastolic and End-Systolic InterVentricular Septum thickness; LVEDD and LVESD: Left Ventricular End-Diastolic and End-Systolic Diameters; LVPW, ED and LVPW, ES: End-Diastolic and End-Systolic Left Ventricular Posterior Wall Thickness. ns, non significant,* Trpm4+/+vs.Trpm4-/; †12 vs. 32 weeks-old mice. * or † P<0.05, ** or †† P<0.01, *** or ††† P<0.001. (DOCX) [file pone.0115256.s007.docx]

**Table S2**: **Left Ventricular basal characteristics in 32 weeks-old *Trpm4^+/+^* and *Trpm4^-/-^* mice.**

|  | *Trpm4^+/+^* |  | | | *Trpm4^-/-^* |  | | | *P value* |  |
| --- | --- | --- | --- | --- | --- | --- | --- | --- | --- | --- |
| Parameters (mm) | ***32 weeks (n=8)*** | | ***P value*** | ***32 weeks (n=7)*** | | | ***P value*** | ***32 weeks*** | | |
| IVS. ED | 0.82 ± 0.03 | | ns | 1.13 ± 0.03 | | | †† | *** | | |
| IVS. ES | 1.08 ± 0.03 | | ns | 1.60 ± 0.04 | | | ††† | *** | | |
| LVEDD | 3.76 ± 0.06 | | ns | 4.36 ± 0.10 | | | †† | *** | | |
| LVESD | 2.98 ± 0.10 | | ns | 2.99 ± 0.14 | | | ns | ns | | |
| LVPW. ED | 0.78 ± 0.03 | | ns | 1.06 ± 0.03 | | | † | *** | | |
| LVPW. ES | 1.00 ± 0.04 | | ns | 1.40 ± 0.03 | | | † | *** | | |

Values are mean ± SEM. IVS,ED and IVS,ES : End-Diastolic and End-Systolic InterVentricular Septum thickness; LVEDD and LVESD: Left Ventricular End-Diastolic and End-Systolic Diameters; LVPW,ED and LVPW,ES : End-Diastolic and End-Systolic Left Ventricular Posterior Wall Thickness. ns, non significant,* *Trpm4^+/+^vs.Trpm4^-/^*; †12 *vs.* 32 weeks-old mice. * or † *P*<0.05, ** or †† *P*<0.01, *** or ††† *P*<0.001.
